# Xu Chunfu’s Modified Xianglian Pill Regulates the NOX2/ROS/Mitochondria/NLRP3 Axis to Treat Ulcerative Colitis

**DOI:** 10.3390/ph19030452

**Published:** 2026-03-11

**Authors:** Shangling Mao, Yuqing Wang, Qingru Bu, Ziyi Xu, Wenfan Wei, Daqiang Wu, Rongfeng Hu, Changzhong Wang, Tianming Wang, Yue Yang

**Affiliations:** 1 College of Integrated Chinese and Western Medicine, Anhui University of Chinese Medicine, Hefei 230012, China; maoshangling0717@163.com (S.M.); wangyuqing202301@163.com (Y.W.); buqingru0703@163.com (Q.B.); 18755428267@163.com (Z.X.); wenfanwei2022@163.com (W.W.); daqwu@126.com (D.W.); ahwcz63@sina.com (C.W.); 2 Key Laboratory of Xin’an Medicine (Anhui University of Chinese Medicine), Ministry of Education, Hefei 230038, China; hurongfeng@163.com

**Keywords:** Xu Chunfu’s Modified Xianglian Pill, ulcerative colitis, NOX2/ROS/mitochondria/NLRP3 axis, gut microbiota

## Abstract

**Background/Objectives**: Xu Chunfu’s Modified Xianglian Pill (XXLP) has been used for centuries in Chinese medicine to treat “diarrhea” and “dysentery,” conditions analogous to modern ulcerative colitis (UC). However, the scientific basis for its efficacy and mechanisms remains unclear. **Methods**: The chemical composition of XXLP was analyzed via UPLC-ESI-MS/MS. A colitis mouse model was established using DSS, and the therapeutic effects were assessed based on body weight, disease activity index (DAI), colon length, and histopathology. Inflammatory cytokines were measured using ELISA. Proteomic analysis and molecular docking identified key targets, which were validated using LPS-induced HT-29 cells via Western blot (WB), qRT-PCR, immunofluorescence (IF), and transmission electron microscopy (TEM). Gut microbiota composition was analyzed using 16S rRNA gene sequencing. **Results**: Analysis of XXLP led to the detection of 373 compounds. XXLP significantly improved colitis symptoms, including weight loss and colon shortening, and reduced the concentrations of inflammatory markers IL-1β, IL-18, TNF-α, and IL-6. Proteomics and molecular docking identified NADPH oxidase 2 (NOX2) as a key target of XXLP intervention in mice with colitis. qRT-PCR, WB, IF, and TEM results further confirmed that XXLP effectively suppressed the expression of NOX2 and its associated protein levels. Sequencing analysis of 16S rRNA showed that XXLP significantly increased the relative abundance of beneficial bacterial genera (*Muribaculaceae* and *Ruminococcaceae*) while markedly reducing the levels of harmful bacteria (*Enterobacteriaceae*). Correlation analysis revealed that specific microorganisms were correlated with NOX2-related protein expression and severity of colonic inflammation. **Conclusions**: XXLP effectively alleviates colitis by suppressing inflammatory responses. Its mechanism involves regulating the NOX2/ROS/mitochondria/NLRP3 axis and altering gut microbiota composition, providing novel insights for colitis treatment.

## 1. Introduction

Ulcerative colitis (UC) is a long-term, advancing inflammatory condition of the bowel that poses a major public health burden worldwide because of its high prevalence and the possibility of irreversible intestinal damage [1,2]. The development of UC is widely attributed to multifaceted interactions involving genetic vulnerability, immune dysregulation, an imbalance in gut microbiota, and compromised intestinal barrier performance [3,4]. Current pharmacological treatments are the mainstay of UC management. First-line therapies typically involve aminosalicylates, such as mesalazine; however, their efficacy as monotherapy is often limited, necessitating their combination with corticosteroids or immunosuppressants. The use of corticosteroids, while effective for acute flares, is constrained by significant side effects, drug resistance, and a lack of evidence for long-term relapse prevention, leading to controversy over their chronic use [5]. Similarly, immunosuppressants are often debated regarding their clinical value and are often limited by their high costs [4]. Thus, the development of new therapies and strategies for more effective management of gastrointestinal symptoms and improvement in outcomes in patients with UC is urgently required.

The nicotinamide adenine dinucleotide phosphate (NADPH) oxidase (NOX) group is a key factor in the generation of reactive oxygen species (ROS). Its member, NOX2, is noted in multiple cell varieties, including intestinal epithelial cells, and is closely implicated in diseases such as UC [6,7]. In its resting state, the NOX2 complex, which consists of membrane components gp91/p22 phox and cytosolic elements p40phox p47phox, p67phox, and Rac GTPase, remains inactive. However, upon stimulation by pathogenic signals, cytosolic proteins such as p47phox become phosphorylated and translocate to bind with the membrane subunits, ultimately activating NOX2 to generate ROS [8,9]. This activation initiates a deleterious cascade of events. ROS produced by NOX2 directly induce mitochondrial dysfunction, and the resulting damaged mitochondria, in turn, produce more ROS, establishing a vicious positive feedback loop that amplifies NOX2 activation and exacerbates intestinal mucosal damage [10,11]. Furthermore, excess ROS and mitochondrial DNA (mtDNA) released from these damaged organelles serve as damage-related molecular patterns (DAMPs) that trigger the NLRP3 inflammasome [12,13]. Consequently, Caspase-1 is activated, splitting the precursors pro-IL-18 and pro-IL-1β into their mature forms. Subsequently, these cytokines work together to produce IFN-γ along with additional inflammatory substances, including TNF-α and IL-6, promoting the permeation of proinflammatory cells [14,15,16]. The self-amplifying inflammatory cascade ultimately results in severe tissue damage, which is characteristic of chronic diseases such as UC. Therefore, therapeutic strategies targeting the NOX2-mitochondria-NLRP3 axis represent a promising avenue for UC treatment.

Inflammatory bowel disease (IBD), which includes UC and Crohn’s disease, is often characterized by upregulated NOX2 expression, abnormally elevated ROS levels, and severe gut microbiota dysbiosis [17,18]. Notably, several IBD risk genes, such as NCF2 and NCF4, encode essential components of the NOX2 complex, underscoring the genetic basis of this association [19]. Under physiological conditions, the gut microbiota and NOX2/ROS system maintain a dynamic balance: the microbiota stimulate moderate ROS production for host defense and signaling, whereas ROS and immune responses regulate the microbiota within a healthy range. However, in pathological states, this equilibrium is disrupted, triggering a vicious cycle. Specifically, dysbiosis leads to excessive activation of NOX2 and overproduction of ROS, which causes tissue damage and compromises the intestinal barrier. This disruption facilitates dysbiosis and sustained immune stimulation [20]. Simultaneously, oxidative stress and microbial metabolites alter mitochondrial metabolism, impairing mitochondrial function [21]. These mitochondrial damage signals, acting synergistically with microbial products such as LPS, serve as key activation cues that initiate NLRP3 inflammasome assembly and activation. Strong pro-inflammatory cytokines, such as IL-1β and IL-18, mature and are released as a result of this process, ultimately driving and amplifying persistent inflammation and tissue damage within the intestinal mucosa [22,23].

**Figure 1:**
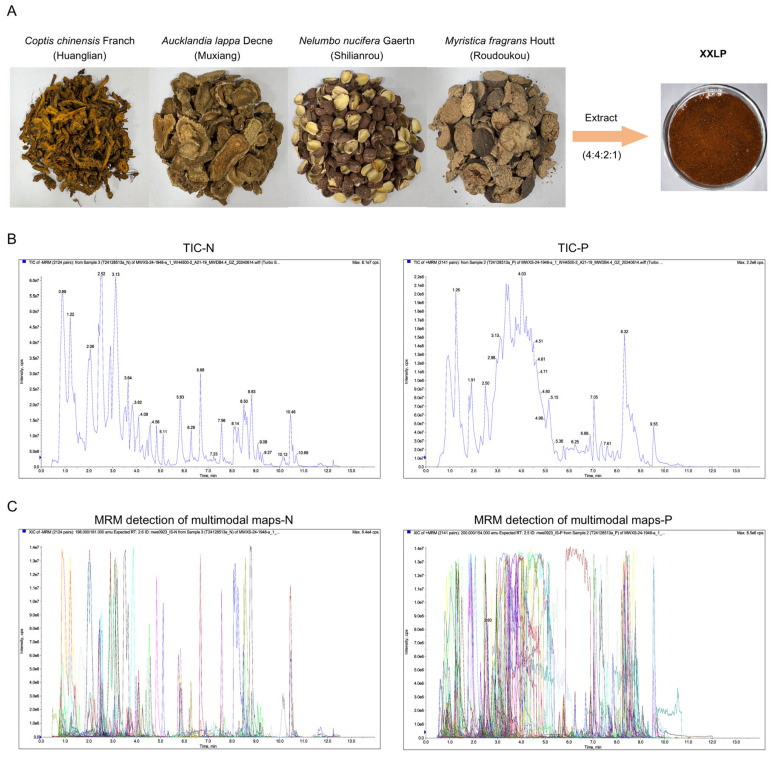
Analysis of XXLP components using UPLC-ESI-MS/MS. (**A**) Composition of the XXLP formula. (**B**) Total ion chromatograms (TICs) of the XXLP. (**C**) Multiple reaction monitoring (MRM) detections for multimodal maps of XXLP. Different colors typically represent different ion pairs or compounds. N and P denote the negative and positive ion modes, respectively.

Xin’an Medicine, a major regional school of traditional Chinese medicine (TCM), originated in the Huizhou region (modern-day Huangshan, Anhui Province) over a millennium ago. Its history traces back to the Northern Song Dynasty, and it reached its peak in the Ming and Qing dynasties. This school is characterized by a distinctive theoretical framework, which includes principles such as “strengthening the body’s constitution and replenishing vital essence”, “restoring Qi and stimulating blood”, “fortifying the spleen to transform dampness”, and “nourishing Yin and unblocking the collaterals” [24]. These established theories provide unique methodologies and extensive clinical experience for managing complex diseases such as UC. Xu Chunfu’s Modified Xianglian Pill (XXLP) is a classical formula documented in the “Medical Complete Book, Ancient and Modern”, a medical book authored by the Xin’an physician Xu Chunfu during the Ming Dynasty. It comprises four herbal ingredients: *Coptis chinensis* Franch. (Huanglian), *Aucklandia lappa* Decne. (Muxiang), *Nelumbo nucifera* Gaertn. (Shilianrou), and *Myristica fragrans* Houtt. (Roudoukou). The ingredients were used in a ratio of 4:4:2:1 (specifically, 60 g: 60 g: 30 g: 15 g), as shown in Figure 1A. The Huanglian-Muxiang pair is often used as an herb pair and forms the famous formula “Xianglian Pill” for treating damp-heat dysentery [25]. Huanglian (monarch drug) clears heat, dries dampness, detoxifies, and stops diarrhea. Muxiang (minister drug) strengthens the stomach, promotes digestion, regulates Qi to alleviate pain, and stops diarrhea. The synergy between these two drugs promotes Qi circulation to resolve stagnation, harmonizes the stomach, fortifies the spleen and intestines, and ultimately ceases dysentery and diarrhea [26]. Roudoukou (assistant drug) promotes Qi circulation and astringes the intestines to stop diarrhea [27]. Shilianrou (envoy drug) tonifies the spleen, nourishes the heart, and calms the mind, thereby providing tonifying and harmonizing functions [28]. For chronic diarrheal disorders, such as dysentery, characterized by deficient righteous qi failing to contain the addition of astringent substances, such as Shilianrou and Roudoukou, moderates the formula’s harshness and promotes recovery.

Modern pharmacological studies have revealed that XXLP contains multiple active components that exert therapeutic effects against UC. The principal herb, Huanglian, exerts potent anti-inflammatory, immunomodulatory, and antibacterial effects, which are largely attributed to its isoquinoline alkaloids [29]. Berberine ameliorates UC by modulating NF-κB and IL-6/STAT3 signaling [30], whereas palmatine inactivates the NLRP3 inflammasome via mitochondrial autophagy [31]. The co-minister herb, Muxiang, contributes costunolide, which directly inhibits NLRP3 activation [32], and dehydrocostuslactone, which restores gut homeostasis by enriching Akkermansia and fortifying the intestinal barrier [33]. Additionally, myristicin from Roudoukou mitigates colitis through the Nrf-2/HO-1 and NF-κB pathways [34], and nuciferine from Shilianrou suppresses inflammation in the gut by preventing NLRP3/Caspase-1 and MAPK/NF-κB signaling [35].

Although XXLP is clinically validated and contains individual compounds with known anti-UC activities, its overall pharmacological mechanism as a holistic formula remains poorly understood. Therefore, this study established a model of DSS-induced UC mice to systematically assess the ameliorating benefits of XXLP and discover its mechanisms of action, with an emphasis on its regulation of inflammation, oxidative stress, and gut microbiota. These findings are expected to provide novel experimental evidence for clarifying the key therapeutic pathways involved in UC.

## 2. Results

### 2.1. Compound Identification and General Overview for XXLP

The constituents of XXLP were thoroughly identified using UPLC-ESI-MS/MS. TIC and MRM profiles of the XXLP sample are shown in Figure 1B,C. A total of 373 compounds were identified, including 51 lignans and coumarins, 50 alkaloids, 47 phenolic acids, 45 amino acids and their derivatives, 39 lipids, 20 nucleotides and derivatives, 18 organic acids, 16 terpenoids, 10 flavonoids, 3 tannins, 2 quinones, and 72 other compounds. The compounds are listed in Supplementary Table S1.

### 2.2. XXLP Treatment Ameliorates Intestinal Inflammation in UC Mice

**Figure 2:**
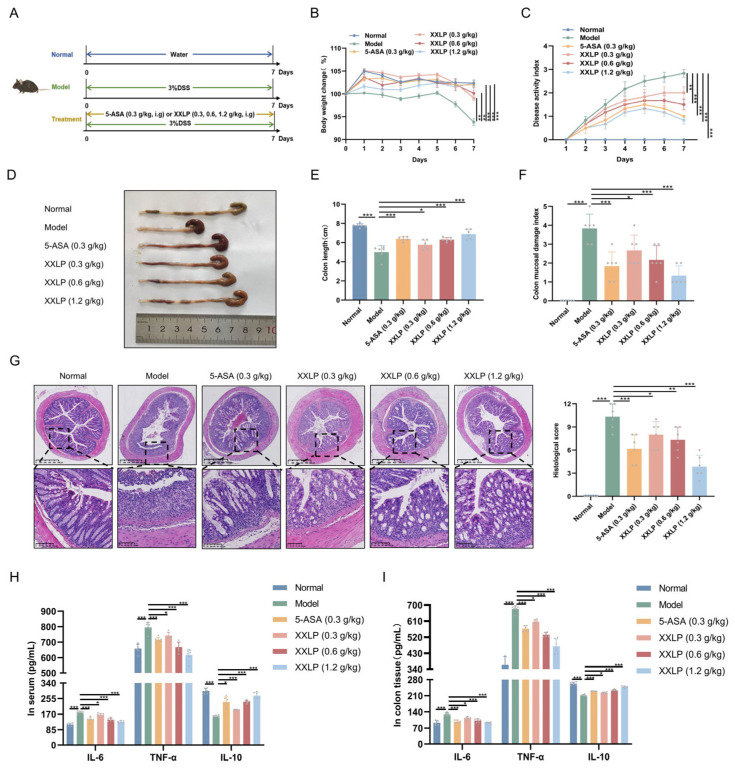
XXLP ameliorated DSS-induced colitis in mice. (**A**) Schematic of the experimental design. (**B**) Changes in body weight. (**C**) DAI score. (**D**) Representative images of colon length. (**E**) Quantitative analysis of colon length. (**F**) CMDI score. (**G**) H&E staining of the colonic tissues. (**H**) Concentrations of IL-6, TNF-α, and IL-10 in serum. (**I**) Concentrations of IL-6, TNF-α, and IL-10 in colon tissues. Data are expressed as mean ± SD (n = 6). * *p* < 0.05, ** *p* < 0.01, *** *p* < 0.001.

The effectiveness of XXLP in UC treatment was evaluated using a DSS-induced mouse model (Figure 2A). Compared to the DSS group, XXLP administration led to a significant dose-dependent reduction in DAI scores. As this integrated score serves as an indicator of clinical severity, encompassing weight loss, diarrhea, and rectal bleeding, the observed decline suggests symptom alleviation (Figure 2B,C). Moreover, XXLP and 5-ASA administration significantly attenuated DSS-induced colon shortening (Figure 2D,E) and reduced CMDI scores (Figure 2F). Histopathological examination revealed that XXLP markedly mitigated mucosal structural damage, attenuated inflammatory cell infiltration, and prevented crypt destruction (Figure 2G). To evaluate the systemic safety of XXLP, we performed histopathological analysis of the liver, kidney, and spleen. The results showed no significant toxic lesions in any of the major organs in the XXLP-treated groups (Supplementary Figure S1). Consistent with these improvements, XXLP administration led to a dose-dependent reduction in the concentrations of proinflammatory mediators IL-6 and TNF-α in both serum and colonic tissue, while reversing the DSS-induced downregulation of interleukin-10 (IL-10) (Figure 2H,I). Collectively, these results demonstrate that XXLP exerts potent therapeutic effects against UC by mitigating multiple pathological changes.

### 2.3. Proteomic Analysis Reveals That XXLP Alleviates UC by Modulating Key Proteins and Pathways

**Figure 3:**
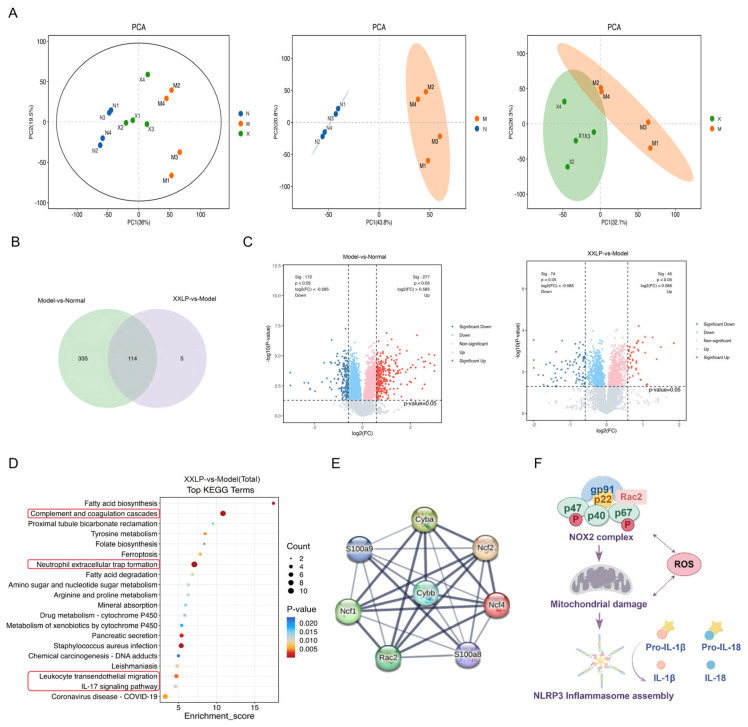
Proteomic analysis based on TMT quantification revealed the mechanism by which XXLP alleviates UC (n = 4). (**A**) PCA analysis. (**B**) Venn diagram analysis. (**C**) Volcano plot. (**D**) KEGG pathway enrichment analysis. (**E**) Protein-protein interaction (PPI) network of the differentially expressed proteins. (**F**) Schematic representation of the interaction networks and downstream mechanisms of the target proteins. N: Normal group; M: Model group; X: XXLP group.

To clarify the molecular pathways responsible for the therapeutic action of XXLP in UC, we employed high-throughput proteomics to systematically analyze colon tissues from control, UC model, and high-dose XXLP (1.2 g/kg) mice. Principal component analysis (PCA) indicated marked spatial clustering among the three groups in a two-dimensional space, suggesting significant differences in the overall protein expression profiles. In contrast to the normal group, the protein level profiles in the model group exhibited marked changes, whereas the high-dose XXLP group showed a trend toward partial reversion to the normal profile (Figure 3A). Using rigorous filtering criteria (FC > 1.5, *p* < 0.05), 454 differentially expressed proteins (DEPs) were identified across the groups (Figure 3B). Comparative analysis revealed 277 upregulated and 172 downregulated proteins in the model group compared to the normal group. In contrast, comparing the XXLP treatment group with the model group revealed 45 upregulated and 74 downregulated proteins (Figure 3C). KEGG pathway enrichment analysis indicated that XXLP significantly regulated multiple signaling pathways associated with inflammation and oxidative stress (Figure 3D). To further investigate the core proteins responsible for these pathway alterations, a protein–protein interaction (PPI) network analysis was conducted. We constructed an interaction network centered on NOX2 (Cybb), highlighting significant interactions with its core subunits p22phox (Cyba), p67phox (Ncf2), p21 (Rac2), p40phox (Ncf4), p47phox (Ncf1), S100A8 (S100a8), and S100A9 (S100a9) (Figure 3E, Table 1). The PPI network identified NOX2 as a central hub, consistent with the KEGG results. This is because the enriched pathways (complement activation, IL-17 signaling, NET formation, and leukocyte migration) all converge on NOX2-mediated ROS production. Given that NOX2 is the primary source of ROS and its activation is a key upstream event in the NLRP3 inflammasome pathway, we propose that the modulation of NOX2 by XXLP is a central mechanism by which XXLP alleviates UC. This proposed mechanism, linking NOX2 activation to NLRP3 inflammasome assembly and subsequent cytokine release, is shown in Figure 3F.

### 2.4. Molecular Docking Analysis of Active Components in XXLP with Subunits of the NOX2 Complex

**Figure 4:**
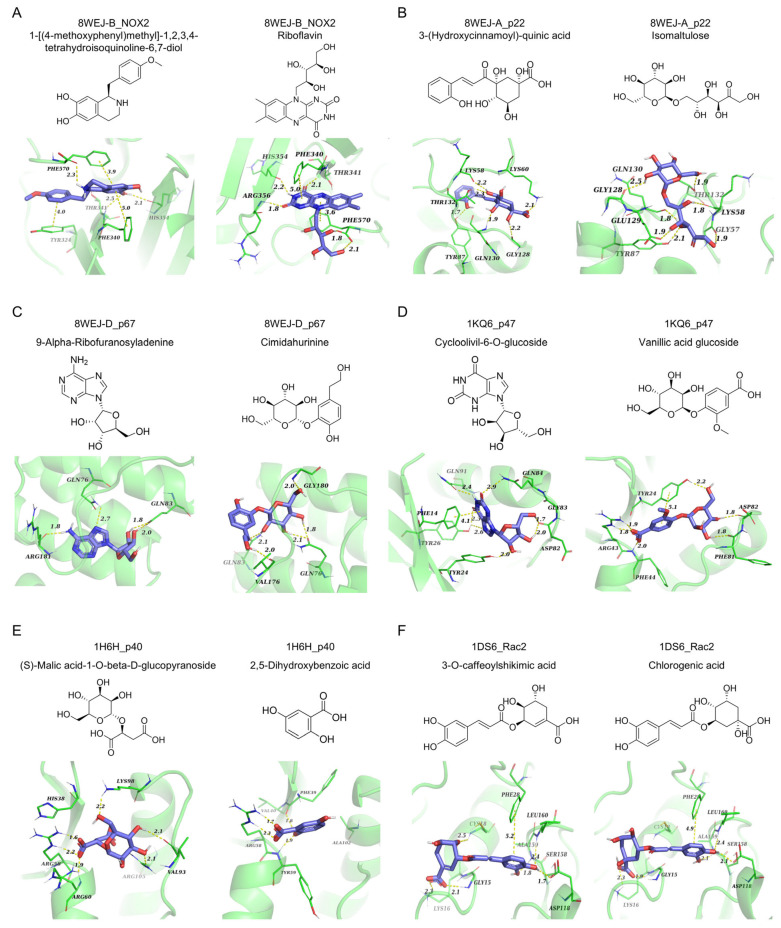
Molecular docking analysis of XXLP compounds with NOX2 and related proteins. The top two compounds exhibiting the strongest affinities for (**A**) NOX2, (**B**) p22, (**C**) p67, (**D**) p47, (**E**) p40, and (**F**) Rac2 are shown.

In molecular docking analysis, the primary active components of XXLP establish specific interactions with key amino acid residues inside the active pocket of the target protein. Specifically,1-[(4-methoxyphenyl)methyl]-1,2,3,4-tetrahydroisoquinoline-6,7- diol interacts with NOX2 via hydrogen bonds with His354, Thr341, and Phe570, while engaging in π-π stacking interactions with Phe340, Phe570, and Tyr324. Riboflavin potently binds to NOX2, creating hydrogen bonds with Phe340, Arg356, His354, and Phe340 and participates in π-π stacking with Phe340 and Phe570 (Figure 4A). 3-(Hydroxycinnamoyl)-quinic acid effectively binds to p22, forming a hydrogen-bond network with Thr132, Lys58, Tyr87, Gln130, and Gly128 and establishing a salt bridge with Lys60. Isomaltulose effectively binds to p22, forming extensive hydrogen bonds with Gly128, Gln130, Glu129, Tyr87, Gly57, Thr132, and Lys58 (Figure 4B). The compound 9-Alpha-Ribofuranosyladenine binds potently to p67, establishing hydrogen bonds with residues Gln83, Gln76, and Arg181, while cimidahurinine effectively binds to p67, exhibiting hydrogen bond interactions with Gly180, Gln76, Val176, and Gln83 (Figure 4C). Cycloolivil-6-O-glucoside effectively binds to p47, binding via hydrogen bonds to Asp82, Gln84, Gln91, Tyr24, and Tyr26, and exhibiting π-π stacking with Phe14. Vanillic acid glucoside binds potently to p47, establishing hydrogen linkages with Tyr24, Asp82, Phe81, and Phe44, forming a salt bridge with Arg43, and exhibiting π-π stacking with Tyr24 (Figure 4D). (S)-Malic acid-1-O-beta-D-glucopyranoside interacts with p40, forming bonds with Arg105, Val93, Lys98, and Arg60, and establishing a salt bridge with Arg58. 2,5-Dihydroxybenzoic acid binds potently to p40, forming hydrogen linkages with Tyr59 and Phe39 and establishing a salt bridge with Arg58 (Figure 4E). Finally, both 3-O-caffeoylshikimic acid and chlorogenic acid were found to associate efficiently with Rac2, establishing four hydrogen linkages with Asp118, Leu160, Gly15, and Ala159, forming a salt bridge with Lys16, and participating in π-π stacking with Phe28. Additionally, 3-O-caffeoylshikimic acid forms an extra hydrogen bond with Cys18. The calculated docking scores for these compounds were −9.629, −9.851, −8.468, −8.782, −8.233, −8.412, −7.387, −7.335, −9.979, −9.98, −8.615, −8.632, respectively (Figure 4F). These interactions collectively reveal the structural basis for compound-target binding, providing crucial insights for subsequent studies.

### 2.5. XXLP Inhibits NOX2 Activation and ROS Production

**Figure 5:**
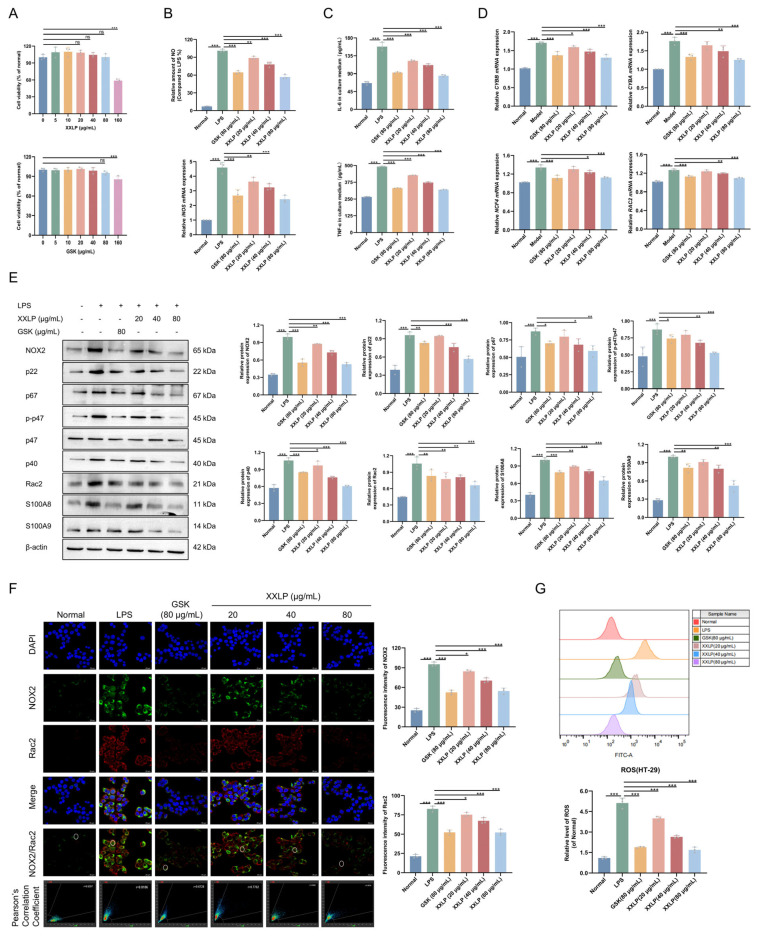
XXLP inhibits the activation of NOX2 and reduces ROS production in vitro. (**A**) Cell viability was assessed using the CCK-8 assay. (**B**) NO levels and mRNA expression of iNOS. (**C**) Concentrations of IL-6 and TNF-α. (**D**) mRNA expression of CYBB, CYBA, NCF4, RAC2. (**E**) Protein expression of NOX2, p22, p67, p-p47, p47, p40 Rac2, S100A8, and S100A9. (**F**) IF staining of NOX2 and Rac2. (**G**) Intracellular ROS levels were detected using flow cytometry. Data are expressed as mean ± SD (n = 3). * *p* < 0.05, ** *p* < 0.01, *** *p* < 0.001. Abbreviation: ns, no significance.

To elucidate whether XXLP mediates its therapeutic effects by regulating NOX2 activation, we established an LPS-induced HT-29 cell inflammation model. CCK-8 assay results showed that 80 μg/mL XXLP and GSK2795039 (a NOX2 inhibitor) had no significant effect on cell viability (Figure 5A). Therefore, 20, 40, and 80 μg/mL XXLP and 80 μg/mL GSK2795039 were selected for subsequent cell experiments. Following XXLP and GSK2795039 intervention in the inflammatory cell model, NO production was significantly reduced, and inducible nitric oxide synthase (iNOS) mRNA expression was markedly downregulated (Figure 5B). In addition, the concentrations of IL-6 and TNF-α were considerably diminished (Figure 5C). Treatment with XXLP notably downregulated the mRNA levels of NOX2 (CYBB) and its associated subunits p22 (CYBA), p40 (NCF4), and Rac2 (RAC2) (Figure 5D). Concurrently, WB analysis confirmed a significant downregulation of NOX2 protein expression and its core subunits, including p22, p47, p67, p40, Rac2, S100A8, and S100A9 (Figure 5E). These results are consistent with previous proteomic sequencing data. IF results further revealed a substantial reduction in the distribution of NOX2 and Rac2 on the cell membrane, indicating effective inhibition of their activation (Figure 5F). Subsequently, a significant reduction in ROS production was observed following XXLP treatment (Figure 5G). These results suggest that XXLP inhibits NOX2 activation by reducing mRNA and protein levels and downregulating key subunits (p22, p67, p47, p40, and Rac2) and related proteins (S100A8/A9). Together, these data show that XXLP effectively blocks NOX2 complex assembly and activation, leading to decreased ROS production.

### 2.6. XXLP Inhibits Mitochondrial Damage and NLRP3 Activation

**Figure 6:**
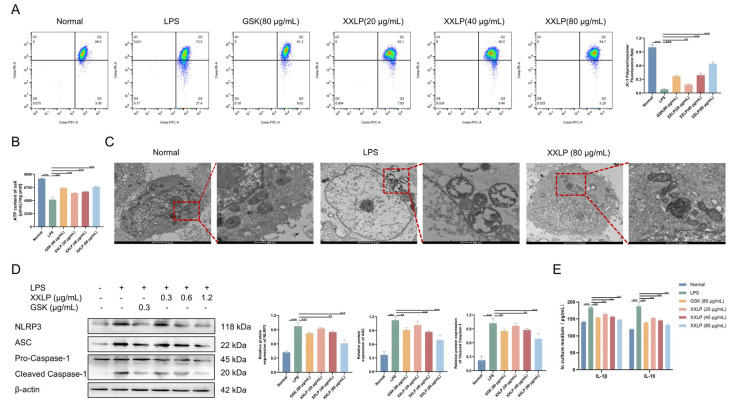
XXLP inhibits mitochondrial damage and NLRP3 activation in vitro. (**A**) Mitochondrial membrane potential was measured using flow cytometry. (**B**) Intracellular ATP level. (**C**) TEM images of mitochondrial integrity. (**D**) Protein expression of NLRP3, ASC, Caspase-1 and Cleaved Caspase-1. (**E**) Concentrations of IL-1β and IL-18. Data are expressed as mean ± SD (n = 3). ** *p* < 0.01, *** *p* < 0.001.

As NOX2-derived ROS can directly or indirectly impair mitochondrial function, we assessed critical parameters, such as MMP, ATP levels, and mitochondrial ultrastructure, to determine whether XXLP attenuates mitochondrial damage. JC-1 staining revealed a significant decrease in the mitochondrial membrane potential in LPS-treated cells, characterized by a reduced red/green fluorescence intensity ratio, indicative of severe mitochondrial depolarization. Following XXLP treatment, the membrane potential was restored in a dose-dependent manner (Figure 6A). Additionally, ATP assays confirmed impaired ATP synthesis in LPS-treated cells, whereas treatment with GSK or XXLP significantly improved ATP production (Figure 6B). TEM revealed that the LPS group exhibited pronounced cellular and mitochondrial swelling and cristae disruption. In contrast, XXLP treatment preserved structural integrity, attenuated swelling, and increased cristae density (Figure 6C).

Both ROS generated by NOX2 complex activation and mitochondrial dysfunction, as well as mtDNA released into the cytosol, can serve as DAMPs that trigger NLRP3 inflammasome activation. To confirm the inhibitory effect of XXLP on this pathway, WB analysis revealed markedly elevated levels of ASC, cleaved caspase-1, and NLRP3 in LPS-treated cells compared to controls, indicating robust inflammasome activation. Conversely, the administration of XXLP led to a marked reduction in the levels of these core essential proteins, exhibiting a dose-dependent pattern (Figure 6D). Furthermore, ELISA assays on cell culture supernatants demonstrated that XXLP potently inhibited LPS-triggered discharge of IL-1β and IL-18(Figure 6E). These results indicate that XXLP ameliorates mitochondrial dysfunction by inhibiting NOX2 complex assembly and ROS production, thereby suppressing downstream NLRP3 inflammasome activation.

### 2.7. XXLP Attenuates Colonic Inflammation in UC Mice via the NOX2/ROS/Mitochondria/NLRP3 Axis

**Figure 7:**
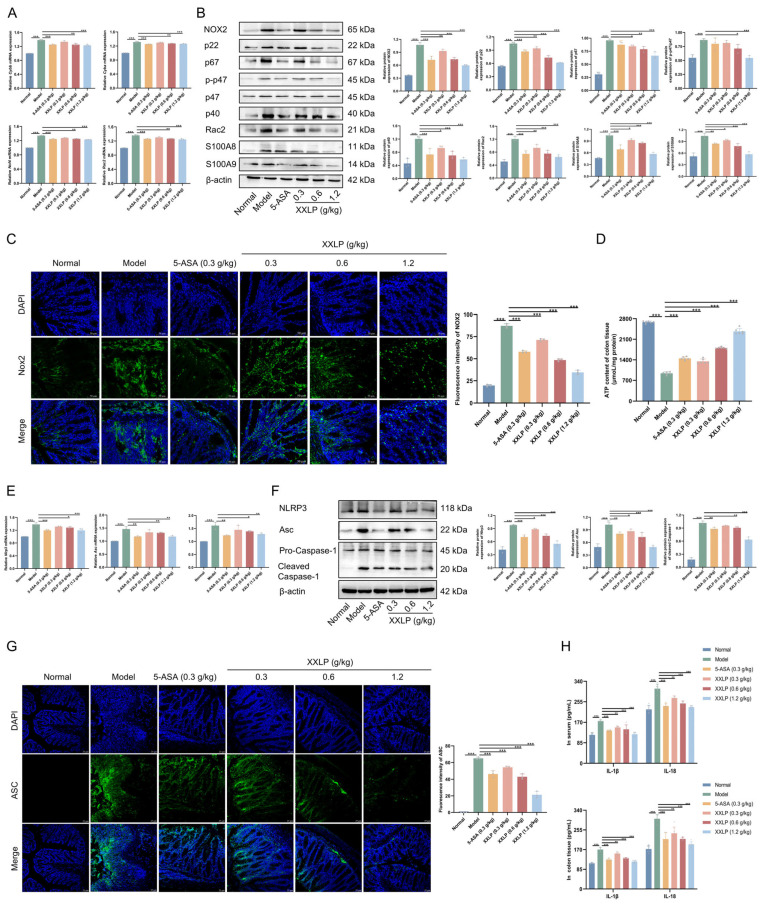
XXLP inhibits the NOX2/ROS/mitochondrial/NLRP3 axis in colon tissues. (**A**) mRNA expression of Cybb, Cyba, Ncf4, and Rac2. (**B**) Protein expression of NOX2, p22, p67, p-p47, p47, p40, Rac2, S100A8, and S100A9. (**C**) IF staining for NOX2. (**D**) ATP levels. (**E**) mRNA expression of Nlrp3, ASC, Caspase-1. (**F**) Protein expression of NLRP3, ASC, Caspase-1 and Cleaved Caspase-1. (**G**) ASC specks stained with anti-ASC antibody. (**H**) Concentrations of IL-1β and IL-18. Data are expressed as mean ± SD (n = 3 for A-C, E-G; n = 6 for D and H). * *p* < 0.05, ** *p* < 0.01, *** *p* < 0.001.

To validate our in vitro findings, we evaluated the involvement of the NOX2/ROS/mitochondria/NLRP3 axis in colonic tissues. Compared to the DSS-administered group, XXLP administration markedly suppressed the mRNA expression of NOX2 (*Cybb*) and its related subunits p22 (*Cyba*), p40 (*Ncf4*), and Rac2, indicating effective transcriptional inhibition (Figure 7A). Consistent with this, WB analysis showed a marked reduction in the expression of NOX2 complex-related proteins (Figure 7B). Furthermore, NOX2 was expressed in colonic tissues, according to IF analysis, and its reduction by XXLP was confirmed by the decreased fluorescence intensity (Figure 7C). Under inflammatory conditions, ATP levels in colonic tissue were significantly depleted; however, following XXLP administration, ATP levels exhibited a dose-dependent recovery, suggesting amelioration of mitochondrial damage (Figure 7D). Further analysis revealed that XXLP administration significantly reduced the activation of the NLRP3 inflammasome, as evidenced by the marked downregulation of both mRNA (Figure 7E) and protein (Figure 7F) expression levels. Concurrently, ASC speck formation was markedly reduced, reflecting an impaired inflammasome assembly (Figure 7G). Moreover, the concentrations of the core inflammation-promoting cytokines IL-1β and IL-18 were markedly diminished in both the serum and colon tissue (Figure 7H). Collectively, these results are consistent with those obtained in the cell model in vitro and provide compelling evidence that XXLP effectively suppresses inflammatory pathway activity by modulating the NOX2-mitochondria-NLRP3 axis, thereby alleviating the pathological progression of UC.

### 2.8. XXLP Changes the Makeup of Gut Bacteria in UC Mice

**Figure 8:**
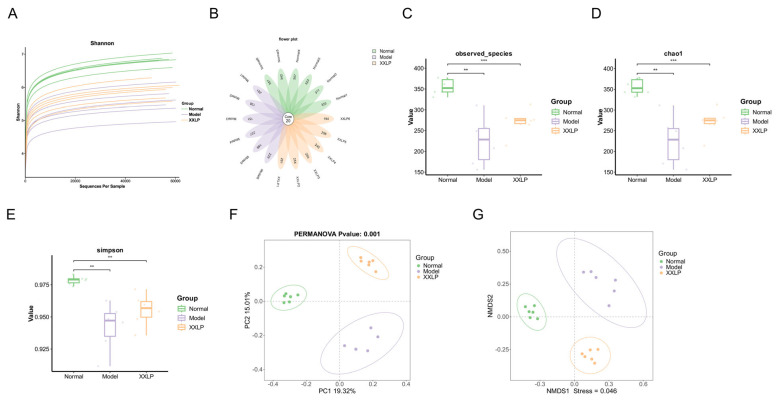
Influence of XXLP on the structure and composition of gut microbiota in DSS-induced UC mice. (**A**) Rarefaction curves of alpha diversity. (**B**) Flower plot displaying the number of ASVs in each sample and the total number of ASVs in all samples. (**C**) Observed species indices. (**D**) Chao1 index. (**E**) Simpson’s index. (**F**) Shannon index. (**G**) NMDS analysis of colonic microbiota based on the unweighted UniFrac metric. ** *p* < 0.01, *** *p* < 0.001.

Gut microbiota dysbiosis plays a crucial role in the pathogenesis of UC. We explored whether the anti-inflammatory actions of XXLP are linked to the modulation of intestinal microbiota. We implemented 16S rRNA amplicon sequencing of the gene, and examination of rarefaction plots revealed that the coverage was adequate to reflect the breadth of microbial variety within the specimens (Figure 8A). A petal diagram illustrated the number of common and exclusive amplicon-derived sequence variants (ASVs) across the Normal, Model, and high-dose XXLP groups, delineating the ASV counts specific to each group and those common to all (Figure 8B). We examined alpha-diversity indices to evaluate the effects of XXLP on microbial diversity and abundance. The Model group showed significantly lower observed species, Chao1, and Shannon indices than the normal group. In contrast, the high-dose XXLP intervention significantly increased these indices (Figure 8C–E), suggesting that XXLP potently improves the diversity and richness of intestinal microflora. Subsequently, beta-diversity analysis was employed to evaluate the similarities and differences among the microbial communities. Principal coordinate analysis (PCoA) (Figure 8F) and non-metric multidimensional scaling (NMDS) (Figure 8G) revealed a distinct distinction between the intestinal microbiota compositions of normal and colitis model mice, indicating divergent community composition patterns. Both analyses demonstrated that DSS significantly altered the gut microbiota structure, whereas XXLP intervention effectively mitigated this disruption.

**Figure 9:**
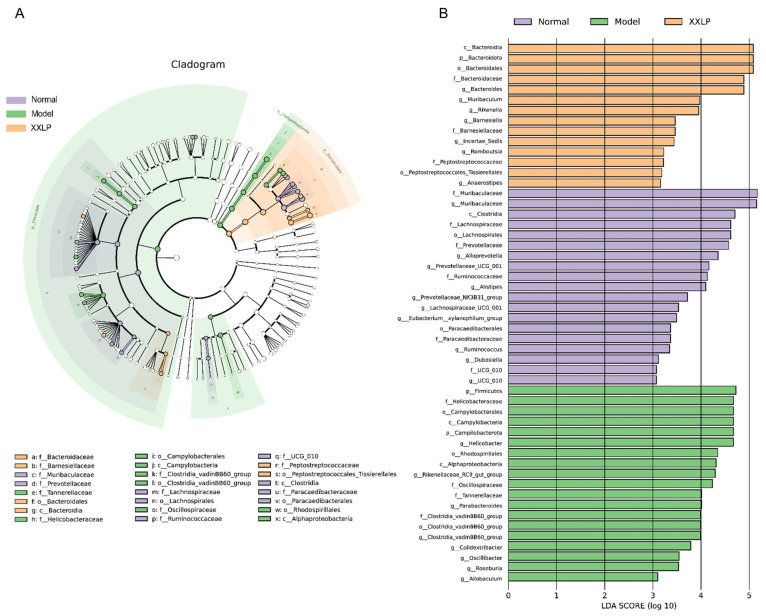
Effect of XXLP administration on the composition of the gut microbiota in mice with DSS-induced colitis (n = 6). (**A**) Taxonomic cladogram (phylum to genus) from LEfSe analysis showing taxonomic associations between groups. Each node represents a specific taxonomic group. (**B**) LDA score distribution for the LEfSe analysis (LDA > 3.5, *p* < 0.05).

**Figure 10:**
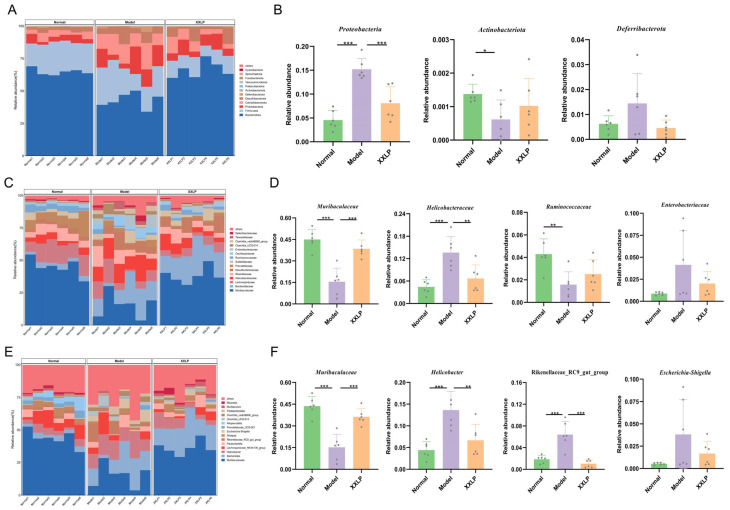
Effect of XXLP on the gut microbiota composition in DSS-induced UC mice. (**A**) Relative abundance of gut microbiota at the phylum level. (**B**) Community composition (bar plot) of the gut microbiota at the phylum level. (**C**) Relative abundance of gut microbiota at the family level. (**D**) Community composition (bar plot) of the gut microbiota at the family level. (**E**) Relative abundance of gut microbiota at the genus level. (**F**) Community composition (bar plot) of the gut microbiota at the genus level. Data are expressed as mean ± SD (n = 6). * *p* < 0.05, ** *p* < 0.01, *** *p* < 0.001.

To further characterize the specific bacterial taxa, LEfSe analysis was performed with an LDA score threshold of >3. The results show that the gut microbiota of the healthy controls was primarily composed of short-chain fatty acid (SCFA)-producing bacteria, such as Muribaculum, Lachnospiraceae, Clostridia, and Ruminococcaceae, indicative of a well-balanced microbiome. Conversely, the model group exhibited significant enrichment of potentially pathogenic and inflammation-associated bacteria, such as Helicobacteraceae, Campylobacterales, and Parabacteroides, which reflects a typical dysbiosis pattern. Following XXLP administration, the gut microbiota composition shifted towards that of the normal group. These results imply that XXLP suppresses the proliferation of pathogenic microorganisms while enhancing the proliferation of probiotic flora, thereby ameliorating the enteric microecological environment (Figure 9). To systematically elucidate changes in microbial composition, we analyzed taxonomic shifts at multiple levels. At the phylum level, Deferribacterota and Proteobacteria exhibited markedly higher relative richness in the DSS group than in the normal group. XXLP intervention effectively reversed these trends (Figure 10A,B). At the family level, XXLP administration markedly diminished the levels of Helicobacteraceae and Enterobacteriaceae in DSS-administered mice, whereas it elevated the levels of Muribaculaceae and Ruminococcaceae (Figure 10C,D). At the genus level, DSS-triggered colitis caused a marked expansion of Helicobacter, Escherichia-Shigella, and Rikenellaceae_RC9_gut_group, establishing them as dominant taxa, whereas the abundance of the beneficial genus Muribaculum was markedly reduced (Figure 10E,F). Collectively, these findings indicate that XXLP restores gut microbiota diversity and structure. Furthermore, by promoting beneficial bacteria and inhibiting pathogenic colonization, XXLP improves microbial functional pathways, providing a mechanistic basis for its therapeutic effect in UC.

### 2.9. Correlation Between Gut Microbiota and Colonic Parameters

**Figure 11:**
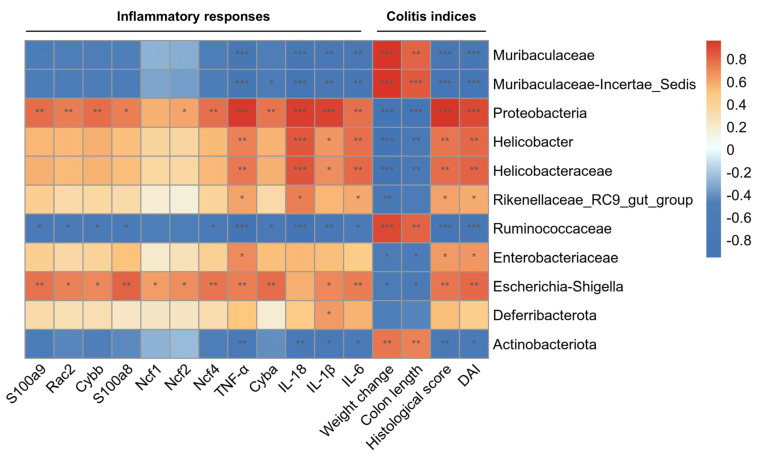
Spearman’s rank correlation analysis illustrating the relationships among gut microbiota, differentially expressed proteins, and colitis symptoms. Positive correlations are shown in red, and negative correlations are shown in blue. Significance levels are indicated as follows: * *p* < 0.05, ** *p* < 0.01, *** *p* < 0.001.

To investigate the association between gut microbiota and UC, we generated Spearman correlation heatmaps mapping specific taxa to colonic parameters and inflammatory responses. The results reveal that certain microbes were positively correlated with colitis symptoms, pathological damage, and inflammatory responses, and negatively correlated with body weight and colon length. For instance, Proteobacteria, Helicobacteraceae, Rikenellaceae-RC9, and Escherichia-Shigella were positively associated with DAI scores, histology scores, and inflammatory indicators, whereas they were negatively associated with body weight and colon length. Furthermore, Proteobacteria, Ruminococcaceae, and Escherichia-Shigella were correlated with the NOX2 complex (Figure 11). These findings indicate that the therapeutic benefits of XXLP in colitis are strongly associated with the regulation of intestinal microbiota.

## 3. Discussion

UC is a long-term idiopathic inflammatory disorder that features a protracted course and recurrent flare-ups, gradually evolving into a globally prevalent and refractory condition [36]. Accumulating evidence suggests that UC progression is closely associated with excessive inflammation and dysbiosis of the gut microbiota [37]. The traditional Chinese medicine formula, XXLP, has been used for hundreds of years to treat gastrointestinal diseases. However, the precise mechanisms responsible for its effectiveness in UC remain unclear. Using combined in vivo and in vitro approaches, we demonstrated for the first time that XXLP potently ameliorates DSS-triggered colitis in mice by suppressing the NOX2/mitochondria/NLRP3 signaling pathway and regulating the intestinal microbiota. Collectively, these findings suggest that XXLP is a promising therapeutic agent for UC management.

NOX2 was the first identified member of the NOX family [38]. To date, it has been extensively reported to be associated with oxidative stress and is considered a primary source of ROS in inflammatory responses [39]. Under physiological conditions, NOX2 participates in pathogen clearance and immune regulation by generating moderate levels of ROS. However, during UC pathogenesis, intestinal epithelial and inflammatory cells infiltrating the intestinal mucosa become excessively activated by stimuli such as LPS and TNF-α, leading to the overactivation of NOX2. This catalyzes the oxidation of NADPH to produce large amounts of superoxide anion (O_2_^−^), which is further converted into highly oxidative ROS [6]. These ROS can directly cause oxidative damage to lipid membranes, proteins, and DNA in intestinal epithelial cells, compromising the intestinal mucosal barrier [40]. They also intensify gut inflammation via the activation of pathways, including MAPK and NF-κB, consequently facilitating the production and secretion of inflammatory cytokines, such as IL-6 and TNF-α [41,42]. Proteomic profiling in the current investigation demonstrated that NOX2 expression was significantly increased in the colon of UC mice relative to controls, whereas XXLP potently suppressed this upregulation. Cell experiments further confirmed that XXLP significantly reduced both NOX2 mRNA and protein levels in the inflammatory cells. We found that XXLP effectively curtails excessive ROS production by inhibiting NOX2 protein activation, exhibiting potency that surpasses that of the inhibitors. Moreover, multiple compounds in XXLP exhibited a strong affinity for NOX2 and its related proteins.

Mitochondrial dysfunction is closely associated with ROS production, and excessive ROS have been confirmed as key inducers of mitochondrial damage, creating a vicious cycle [43]. Specifically, when mitochondria are damaged by NOX2-derived ROS, the electron transport chain (ETC) malfunctions, leading to further ROS production. This amplifies inflammatory signals and forms a positive feedback loop involving activated NOX2, which exacerbates intestinal mucosal injury [44]. Our assessment of the mitochondrial membrane potential and transmission electron microscopy (TEM) demonstrated that XXLP effectively modulated mitochondrial dysfunction. These findings indicate that XXLP reduces excessive ROS production by inhibiting NOX2 activation, thereby effectively mitigating oxidative stress-induced mitochondrial damage. Consequently, this alleviation of mitochondrial damage improves ETC function and decreases ROS accumulation. Impaired mitochondrial function is widely accepted as a pivotal upstream trigger for the activation of the NLRP3 inflammasome [45,46]. Injury disrupts ETC function, resulting in abnormal ROS generation that triggers NLRP3 and fosters the release of inflammation-promoting mediators IL-1β and IL-18 [47]. In UC, this process disrupts the intestinal epithelial barrier and perpetuates inflammation. Our experimental results systematically analyzed NLRP3 protein expression, assembly, and cytokine levels, revealing that XXLP intervention significantly reduced NLRP3 expression, inhibited its assembly, and decreased IL-1β and IL-18 release, while improving ATP synthesis. These findings align with our TEM observations of improved mitochondrial architecture, indicating that XXLP effectively blocks NLRP3 inflammasome activation by alleviating oxidative stress and mitochondrial dysfunction. By protecting mitochondrial function through inhibition of the NOX2/ROS axis, XXLP interferes with NLRP3 assembly and activation. This disrupts the vicious cycle, reduces excessive proinflammatory cytokine release and inflammatory cell infiltration, and ultimately decreases pathological damage to colonic tissue, providing a novel mechanistic explanation for the treatment of UC.

Extensive research indicates that gut microbiota dysbiosis promotes the onset and progression of UC, often accompanied by excessive inflammation [48]. Butyric acid, which is the main energy source for intestinal epithelial cells, downregulates pro-inflammatory factor expression and suppresses inflammation by inhibiting the NF-κB signaling cascade [49]. Ruminococcaceae, a butyrate-producing genus, exhibits a significantly reduced abundance in patients with IBD [50]. Similarly, Muribaculaceae, a commensal bacterium predominant in healthy individuals, primarily participates in butyric acid and tryptophan metabolism [51]. Additionally, Lachnospiraceae exert anti-inflammatory effects by generating short-chain fatty acids, thereby alleviating colitis symptoms [52]. Our results showed that XXLP increased the abundance of certain beneficial bacteria that produce SCFAs, including Muribaculaceae, Ruminococcaceae, and Lachnospiraceae [53]. Spearman correlation analysis revealed that Ruminococcaceae and Muribaculaceae were positively associated with colon length and body weight changes, while negatively correlating with DAI scores and inflammation severity. Conversely, Proteobacteria are commonly associated with the development of intestinal diseases, such as UC, and their overgrowth exacerbates mucosal injury and dysbiosis [54]. The reduction in Helicobacteraceae noted in our study might be connected to the repression of Helicobacter-associated pathogens, thereby lowering the risk of intestinal inflammation [55]. The decrease in Enterobacteriaceae reflects the regulatory effects on potential pathogens, such as Escherichia coli, contributing to the mitigation of dysbiosis and mucosal injury [56]. Furthermore, the pro-inflammatory pathogen Escherichia-Shigella, often detected in patients with IBD, exhibited increased abundance in the DSS-induced UC model [57,58]. Studies have indicated that Escherichia-Shigella correlates positively with inflammation and colitis severity, which is consistent with our findings. Our results demonstrated that Proteobacteria, Helicobacteraceae, Enterobacteriaceae, and Escherichia-Shigella negatively correlated with colon length and body weight but positively correlated with inflammatory factors. Importantly, XXLP treatment downregulated the abundance of these pathogenic bacteria. Collectively, these findings indicate that administering XXLP to UC mice considerably reduces gut microbial dysbiosis. By enhancing the abundance of beneficial bacteria and suppressing harmful bacteria, XXLP effectively restored the intestinal microecological balance.

Although this study elucidates the mechanism by which XXLP modulates the inflammatory cascade in UC by targeting the NOX2 complex and regulating gut microbiota, certain limitations remain. The experimental model primarily employed in mouse UC model to simulate the pathological process may fail to fully recapitulate the clinical heterogeneity and intricacy of the human intestinal microenvironment. In future research, we intend to explore the synergistic effects of XXLP with other emerging therapies, such as gut microbiota modulation and mitochondria-targeted antioxidants, to enhance intestinal epithelial barrier repair. In addition, although XXLP has a complex composition and exhibits multi-targeted characteristics, which poses challenges for mechanistic elucidation, our proteomic analysis provides a data-driven foundation for understanding its therapeutic effects. To align further with modern trends in precision medicine, we intend to conduct further research on bioactive component isolation, structural modification, and specific target identification. These steps will enhance the specificity and predictability of the formulation, bridging the gap between traditional holistic applications and modern targeted drug discovery.

## 4. Materials and Methods

### 4.1. Chemicals and Reagents

DSS (4000 kDa) was acquired from Macklin (Shanghai, China). Mesalamine (5-aminosalicylic acid, 5-ASA, Lot.231215) and used in animal experiments has been bought from the sunflower Pharmaceutical Group Co., Ltd. (Harbin, China). Lipopolysaccharide (LPS) used for cell experiments and the NOX2 inhibitor (GSK2795039) were respectively sourced from Sigma-Aldrich (St. Louis, MO, USA) and MedChem Express (Monmouth Junction, New Jersey, USA). Jianglai Biology (Shanghai, China) supplied the ELISA kits designed for detecting TNF-α, IL-18, IL-1β, IL-6, and IL-10 in both mouse and human samples. Luminescent ATP Detection Assay Kit was purchased from APExBIO (Houston, USA). A kit for detecting reactive oxygen species and mitochondrial membrane potential (JC-1 method) was supplied by Bestbio Biotechnology (Shanghai, China). SYBR Green Master Mix was purchased from Yeasen Biotech (Shanghai, China). CCK-8 reagent kit, Tissue/Cell RNA Rapid Extraction Kit, and RIPA lysis solution were acquired from SparkJade Biotechnology (Shandong, China). Primary antibodies for NLRP3 and Caspase-1 were supplied by Cell Signaling Technology (Danvers, MA, USA). Primary antibodies against NOX2, p22, p47, p67, p40, S100A8, S100A9, ASC, and Cleaved Caspase-1 were obtained from Abclonal (Wuhan, China). Rac2 antibody was obtained from Proteintech (Wuhan, China). The working solution ratios of each antibody are provided in Supplementary Table S2.

### 4.2. Preparation of the XXLP Extract

Coptis chinensis Franch. (Huanglian, Lot. 20221101), Aucklandia lappa Decne. (Muxiang, Lot. 221001), and Nelumbo nucifera Gaertn. (Shilianrou, Lot. 20220101), and Myristica fragrans Houtt. (Roudoukou, Lot. 220301) (4:4:2:1) were procured from the First Affiliated Hospital of Anhui University of Traditional Chinese Medicine (Hefei, China) and identified by Prof. Ning Li, Anhui Medical University. A voucher specimen (No. 20240226) was archived at the Laboratory of the Scientific Research and Experiment Center, College of Integrated Chinese and Western Medicine, Anhui University of Chinese Medicine. The powdered herbs (4:4:2:1) were extracted twice with 95% alcohol (1:8, w/v) under reflux, with each extraction lasting 30 min. The residue was then extracted twice with deionized water (1:8, *w*/*v*) under reflux. The merged filtrates were concentrated and freeze-dried, and the resulting powder was stored at 4 °C until further use.

### 4.3. Identifying the Components of XXLP Through UPLC-MS/MS

The experimental procedure was conducted as described previously [59].

### 4.4. Animal Experiments

Thirty-six male C57BL/6 mice (6–8 weeks old, 20–22 g) were obtained from Hangzhou Ziyuan Laboratory Animal Technology Co., Ltd. and housed in compliance with SPF standards. All animal experiments were approved by the Animal Ethics Committee of Anhui University of Chinese Medicine and performed in accordance with Institutional Animal Care and Use Committee (IACUC) guidelines (Approval No. AHUCM-mouse-2024233). Mice were randomly assigned to the following groups (n = 6): Normal, Model, 5-ASA (0.3 g/kg), and XXLP (0.3, 0.6, and 1.2 g/kg). Mice in the Model group were administered 3% DSS in their drinking water for a week, whereas the normal group received regular water. On day 8, the mice were processed, and blood and colon tissue samples were collected for subsequent analysis. Daily observations were made regarding shifts in fecal blood, stool texture, and body weight. DAI and CMDI were calculated to determine the severity of colitis [60]. We performed macroscopic examination of the colon to assess inflammation and ulceration [61]. In addition, injury to the colonic mucosa was assessed using hematoxylin and eosin (H&E) staining and histological grading [62].

### 4.5. Enzyme-Linked Immunosorbent Assay (ELISA)

Biological specimens, including serum, colon tissue homogenates, and cell culture supernatants, were prepared for cytokine analysis. Briefly, colon tissues (~100 mg) were homogenized in ice-cold PBS and centrifuged at 1500× *g* for 20 min at 4 °C. The resulting centrifugate was then collected. The concentrations of IL-10, IL-6, IL-18, IL-1β, and TNF-α in these specimens were quantified using kits according to the manufacturer’s guidelines.

### 4.6. Proteomics Analysis

Colon tissue samples were pulverized in liquid nitrogen, and 800 μL of phenol extraction solution with phosphatase and protease inhibitors was added. The tissue was homogenized using a cold grinder and then extracted with phenol-Tris-HCl (pH 7.8) solution. The mixture was centrifuged to collect the upper phenolic phase. Total protein was precipitated with ammonium acetate-methanol, dried, re-dissolved in lysis buffer, and cleaned with acetone and methanol. After quantifying the protein content using the BCA assay, a suitable volume of the sample was used for SDS-PAGE validation. Subsequently, 50 μg of protein solution was reduced with DTT, alkylated with iodoacetamide, and precipitated with acetone. Digestion with trypsin was performed at 37 °C for 12 h. The resulting peptide mixture was labeled using TMT reagents, and the reaction was terminated with hydroxylamine after 1 h. The labeled peptides were fractionated using high-pH reversed-phase chromatography (C18 column, pH 10), separated by nano-flow liquid chromatography (C18 column, acidic gradient), and finally analyzed by mass spectrometry. Data-dependent acquisition mode was employed for both primary and secondary mass spectrometry detection to obtain qualitative and quantitative protein data.

### 4.7. Molecular Docking

Ligand preparation was performed using the LigPrep module within the Schrödinger Suite 2025-2, applying the OPLS4 force field to create all probable ionization states and tautomers at pH 7 ± 2. The minimized ligand conformations were docked into the protein structure (PDB ID: 8WEJ-A, 8WEJ-B, 8WEJ-D, 1KQ6, 1H6H, and 1DS6) using Glide within the Schrödinger software, following standard precision (SP) docking protocols to identify the most favorable binding poses. All identified compounds were subjected to docking screening, and the top two compounds with the lowest binding energies were selected for visualization.

### 4.8. Cell Culture

HT-29 human colon cancer cells were obtained from the Chinese Academy of Sciences Cell Bank (Shanghai, China). Cells were grown in McCoy’s 5A complete medium containing 10% FBS under 5% CO_2_ at 37 °C.

### 4.9. CCK-8 Assay

Cellular activity was determined using the CCK-8 method. A density of 5 × 10^3^ HT-29 cells per 100 μL in 96-well plates and incubated for 24 h to facilitate growth. Subsequently, the cultures were exposed to varying doses of XXLP or the NOX2 inhibitor (GSK2795039), ranging from 0 to 160 μg/mL. Then, 10 μL of the CCK-8 reagent was added to each well and incubated for 2 h at 37 °C. Finally, absorbance at 450 nm was detected using a spectrophotometric microplate reader.

### 4.10. Nitric Oxide (NO) Assay

Nitric oxide levels in the cell culture supernatant were quantified using the Griess assay with an NO Detection Kit (Beyotime Biotechnology, Shanghai, China). After collecting the cell lysate, 50 μL of each sample or standard was transferred to the culture plate. Subsequently, equal volumes of Griess Reagents I and II were added sequentially. After a 10 min incubation at 37 °C in the dark, the absorbance at 540 nm was measured using a microplate spectrophotometer. NO levels were calculated by comparison with a standard curve generated from sodium nitrite.

### 4.11. Immunofluorescence (IF)

Sections of colon tissue fixed in paraffin were processed for dewaxing and dehydration, and underwent heat-mediated antigen retrieval. Cells receiving treatment were fixed using a cell fixative for 15 min and then treated with PBS. After a 1 blocking step using 5% goat serum, the specimens were incubated with primary antibodies overnight at 4 °C. The following day, the samples were washed with PBS and treated with fluorescent secondary antibodies for 1 h. DAPI was used for counterstaining the cell nuclei, and the cells were mounted using an anti-fluorescence mounting medium. Images of immunofluorescent staining were acquired using a Leica Stellaris 5 confocal laser scanning microscope (Leica Microsystems, Wetzlar, Germany).

### 4.12. Real-Time PCR Analysis

RNA was extracted from both colonic tissues and cells using an RNA rapid extraction kit, following the manufacturer’s guidelines. Following reverse transcription to generate cDNA, RT-qPCR was performed to assess gene expression. All primer sequences were obtained from Sangon Biotech Co., Ltd. (Shanghai, China). (Supplementary Table S3).

### 4.13. Western Blotting (WB)

The experimental procedure was conducted as previously described [59].

### 4.14. ATP Assay

ATP levels in the colon tissue and cells were measured using a commercial ATP assay kit. ATP Lysis Buffer was used to disrupt fresh tissue samples on ice, and cultured cells underwent the same lysis procedure. Supernatants were obtained by centrifugation. Each well of a black 96 well microplate received 100 μL of ATP working reagent, followed by a 5 min incubation. Subsequently, 20 μL of either supernatant or standard was added, mixed well, and the luminescent intensity was detected immediately.

### 4.15. ROS Assay

After 24 h of treatment, the cells were rinsed with PBS, detached using trypsin, and harvested via centrifugation. The liquid was removed, and the cellular pellet underwent resuspension in the O06 probe working solution (diluted 1:1000 in serum-free medium) from a Cellular ROS Detection Kit (Bestbio Biotechnology, Shanghai, China). The cells were maintained at 37 °C for 20 min with gentle agitation every 5 min. Cells without staining were used as the negative controls. After incubation, the cultures were washed three times with serum-free medium to remove unbound probes. Intracellular ROS concentrations were analyzed by flow cytometry, and FlowJo version 10.8.1 was utilized to perform the data analysis.

### 4.16. Mitochondrial Membrane Potential (MMP) Assay

The JC-1 assay was employed to evaluate changes in mitochondrial membrane potential following the manufacturer’s guidelines (Bestbio Biotechnology, Shanghai, China). Briefly, 24 h post-treatment, cells were collected, rinsed twice with PBS, and pelleted by centrifugation. Resuspended in JC-1 working solution and incubated for 20 min at 37 °C. Centrifuge to collect cells, wash with ice-cold PBS to eliminate excess dye and resuspended in PBS. Fluorescence intensity was detected by flow cytometry, with data analysis conducted using FlowJo 10.8.1 software.

### 4.17. Transmission Electron Microscope (TEM)

The experimental procedure was conducted as previously described [63].

### 4.18. 16S rRNA Sequencing

Collect fresh mouse feces and store in liquid nitrogen until further analysis. 16S rRNA gene sequencing to assess structural variation in the gut microbiota (Oebiotech, Shanghai, China). ASV/OTU data were utilized to calculate alpha-diversity indices, while beta-diversity was assessed using Non-metric Multidimensional Scaling (NMDS) based on the Bray-Curtis dissimilarity metric. QIIME2 software was utilized to analyze taxonomic abundance at the phylum, family, genus, and species ranks. Furthermore, Linear Discriminant Analysis Effect Size (LEfSe) was applied to pinpoint notable variations in the composition of the microbial community between the groups. A two-tailed Pearson correlation test was conducted via R software to compute the correlation coefficient r and its associated statistical significance *p*-value. Present significant correlations (*p* < 0.05) as a heatmap, where color intensity represents the magnitude of the correlation coefficient, visually illustrating the potential relationship between gut microbiota changes and key experimental indicators.

### 4.19. Statistical Analyses

Each experiment was performed independently with no fewer than three repetitions. Data are presented as mean ± SD. IBM SPSS Statistics 27.0 and GraphPad Prism 9.5 were utilized to perform statistical evaluations. For comparing two groups of unpaired independent continuous variables, the unpaired Wilcoxon signed-rank test or Student’s t-test was adopted. Kruskal-Wallis H test combined with one-way ANOVA was utilized to compare multiple groups of fully randomized continuous variables. The false discovery rate (FDR) was managed using the Benjamini-Hochberg method. Significance: * *p* < 0.05, ** *p* < 0.01, *** *p* < 0.001.

## 5. Conclusions

XXLP effectively alleviates colitis by suppressing inflammatory responses. Its therapeutic mechanism involves modulating the NOX2-mitochondria-NLRP3 axis and restoring gut microbiota homeostasis. The present investigation offers new insights into the pharmacological underpinnings of XXLP in treating colitis.

## Figures and Tables

**Table 1:** NOX2 related interacting proteins.

Gene	Protein		Model-vs-Normal			XXLP-vs-Model	
		log2FoldChange	FoldChange	Regulation	log2FoldChange	FoldChange	Regulation
Cybb	gp91	1.47574	2.781262664	Up	−0.65813	0.633699156	Down
Cyba	p22	1.3873625	2.615999926	Up	−0.570875	0.673208361	Down
Ncf1	p47	0.6425075	1.561039994	Up	−0.4592225	0.727378153	Down
Ncf2	p67	0.74185	1.672318916	Up	−0.4503225	0.731879225	Down
Ncf4	p40	1.044595	2.062787202	Up	−0.651005	0.636836531	Down
Rac2	Rac2	1.1124	2.162050169	Up	−0.616405	0.652294333	Down
S100a8	S100a8	2.770965	6.825643192	Up	−1.4019375	0.378420592	Down
S100a9	S100a9	3.0728825	8.414528886	Up	−1.6271925	0.323717553	Down

## Data Availability

The original contributions presented in this study are included in the article/Supplementary Material. Further inquiries can be directed to the corresponding author(s).

## Supplementary Materials

The following supporting information can be downloaded at: https://www.mdpi.com/article/10.3390/ph19030452/s1, Supplementary Figure S1: H&E staining of liver, spleen, and kidney tissues; Table S1: Antibody dilutions; Table S2: Primers used in the quantity real-time PCR assay; Table S3: Primers used in the quantity real-time PCR assay.

## Abbreviations

The following abbreviations are used in this manuscript:

BCA	bicinchoninic acid
CMDI	colon mucosal damage index
DSS	sodium dextran sulfate
DAI	Disease Activity Index
MRM	Multiple Reaction Monitoring
NOX2	NADPH oxidase 2
NCF1	neutrophil cytosolic factor 1
NCF2	neutrophil cytosolic factor 2
NCF4	neutrophil cytosolic factor 4
Rac2	Ras-Related C3 Botulinum Toxin Substrate 2
TNF-α	Tumor Necrosis Factor-alpha
TIC	Total Ion Chromatogram
UPLC-ESI-MS/MS	Ultra-Performance Liquid Chromatography Electrospray Ionization-Tandem Mass Spectrometry
