# Supplementary material for: Xu Chunfu’s Modified Xianglian Pill Regulates the NOX2/ROS/Mitochondria/NLRP3 Axis to Treat Ulcerative Colitis
Source: Pharmaceuticals (Basel). 2026 Mar 11;19(3):452. doi: 10.3390/ph19030452 (PMC13029697; doi:10.3390/ph19030452)
Supplement: Supplementary file 1 [file pharmaceuticals-19-00452-s001.zip › pharmaceuticals-4158266-Table S1.pdf]

Supplementary Table S1 Components of XXLP were identified using a UPLC-ESI-MS/MS system.

Compounds	CAS	Formula	Q1 (Da)	Q3 (Da)	Molecular weight (Da)	Ionization model	Relative Peak Areas
γ-Glutamyl tyramine	65520-56-1	C13H18N2O4	267.13	121.06	266.1267	[M+H]+	1304015.976
γ-Glu-Phe*	7432-24-8	C14H18N2O5	295.13	120.08	294.1216	[M+H]+	152697.196
Xanthurenic Acid 8-O-Glucoside	97451-32-6	C16H17NO9	366.08	160.04	367.0903	[M-H]-	743753.558
Xanthosine	146-80-5	C10H12N4O6	283.07	151.03	284.0757	[M-H]-	342253.733
Vanillin; 4-Hydroxy-3-Methoxybenzaldehyde	121-33-5	C8H8O3	151.04	136.02	152.0473	[M-H]-	22874470.22
Vanillic acid glucoside	32142-31-7	C14H18O9	329.09	167.03	330.0951	[M-H]-	8235959.63
Vanillic acid	121-34-6	C8H8O4	167.03	108.02	168.0423	[M-H]-	3312347.364
Val-Pro	20488-27-1	C10H18N2O3	215.14	116.07	214.1317	[M+H]+	360764.847
Val-Ala-Ile	-	C14H27N3O4	302.21	143.12	301.2002	[M+H]+	315476.716
Urolignoside*	131723-83-6	C26H34O11	521.2	359.15	522.2101	[M-H]-	283712.008
Uridine 5'-monophosphate	58-97-9	C9H13N2O9P	323.03	211	324.0359	[M-H]-	287001.174
Uridine	58-96-8	C9H12N2O6	243.06	110.02	244.0695	[M-H]-	208242.658
Undecanedioic acid	1852-04-6	C11H20O4	215.13	153.13	216.1362	[M-H]-	840248.306
Tyrosylleucine	17355-10-1	C15H22N2O4	295.16	136.08	294.158	[M+H]+	890791.013
Trimethyllysine	23284-33-5	C9H20N2O2	189.16	84.08	188.1525	[M+H]+	536911.407
Trigonelline	535-83-1	C7H7NO2	138.06	94.07	137.0477	[M+H]+	17779809.16
Tributyl acetylcitrate	77-90-7	C20H34O8	403.23	129.02	402.2254	[M+H]+	190789.338
Trehalose 6-phosphate	4484-88-2	C12H23O14P	421.08	241.01	422.0825	[M-H]-	77426.826
Trehalosamine	-	C12H23NO10	342.14	162.07	341.1322	[M+H]+	2521028.89
Tortoside A*	-	C28H36O13	579.21	417.15	580.2156	[M-H]-	642421.899
Theviridoside	23407-76-3	C17H24O11	403.12	167.03	404.1319	[M-H]-	122703.008
Thalmelatine	-	C40H46N2O8	683.33	342.18	682.3254	[M+H]+	40885236.99
Thaliporphine	5083-88-5	C20H23NO4	342.17	297.11	341.1627	[M+H]+	25337191.82
Thalictrifoline	79082-02-3	C21H23NO4	354.17	339.15	353.1627	[M+H]+	10593367.89
Thalicsessine	-	C22H27NO4	370.2	206.12	369.194	[M+H]+	8286095.413
Thalbaicalidine	-	C21H25NO5	372.18	222.11	371.1733	[M+H]+	40726478.49
tetrahydrofuroguaiacin B*	131829-51-1	C20H24O5	345.17	137.06	344.1624	[M+H]+	25919882.5
Tetrahydrocorysamine*	32043-26-8	C20H19NO4	338.14	323.12	337.1314	[M+H]+	52812128.02
Terephthalic acid	100-21-0	C8H6O4	165.02	121.03	166.0266	[M-H]-	368660.187
Syringin	118-34-3	C17H24O9	371.13	209.08	372.142	[M-H]-	246741.994
Syringic acid	530-57-4	C9H10O5	197.05	123.01	198.0528	[M-H]-	849614.135
Syringaresinol Diglucoside	-	C34H46O18	801.28	417.16	742.2684	[M+CH3COO]-	327311
Syringaresinol	21453-71-4	C22H26O8	417.16	402.1	418.1628	[M-H]-	257921.002
Syringaldehyde; 4-Hydroxy-3,5-Dimethoxybenzaldehyde	134-96-3	C9H10O4	181.05	151	182.0579	[M-H]-	16863559.38
Succinyladenosine	4542-23-8	C14H17N5O8	384.12	252.07	383.1077	[M+H]+	2914495.307
Succinic acid*	110-15-6	C4H6O4	117.02	73.03	118.0266	[M-H]-	23361549.55
Suberic Acid	505-48-6	C8H14O4	173.08	111.08	174.0892	[M-H]-	74929.719
Styrylamine	83148-11-2	C8H9N	120.08	77.04	119.0735	[M+H]+	14078803.18

Stachyose	470-55-3	C24H42O21	665.21	341.11	666.2219	[M-H]-	238086.878
sorbose*	3615-56-3	C6H12O6	179.0556	59.0139	180.0634	[M-H]-	19596096.11
Sinapyl alcohol	537-33-7	C11H14O4	209.08	179.07	210.0892	[M-H]-	215104
Sinapinaldehyde	4206-58-0	C11H12O4	207.07	192.04	208.0736	[M-H]-	37599940.38
Shikimic acid	138-59-0	C7H10O5	173.05	93.03	174.0528	[M-H]-	813640.033
Senkyunolide C*	-	C12H12O3	205.09	149.02	204.0786	[M+H]+	6278338.96
Sedoheptulose	3019-74-7	C7H14O7	209.07	59.01	210.074	[M-H]-	216025.153
Sebacic acid	111-20-6	C10H18O4	201.11	139.11	202.1205	[M-H]-	12755.634
S-(5'-Adenosy)-L-homocysteine	979-92-0	C14H20N6O5S	385.13	250	384.1216	[M+H]+	51408.225
Riboflavin (Vitamin B2)	83-88-5	C17H20N4O6	377.15	243.09	376.1383	[M+H]+	1038165.428
Reticuline*	485-19-8	C19H23NO4	330.17	192.1	329.1627	[M+H]+	2453096.629
Raffinose*	512-69-6	C18H32O16	503.1618	179.0551	504.169	[M-H]-	1149385.129
PyroGlu-Ile	-	C11H18N2O4	241.12	197.13	242.1267	[M-H]-	33204.557
Pyrocatechol	120-80-9	C6H6O2	109.03	81.03	110.0368	[M-H]-	211172.385
Pyridoxine	65-23-6	C8H11NO3	170.08	134.06	169.0739	[M+H]+	4863964.766
pseudocodeine*	466-96-6	C18H21NO3	300.16	269.12	299.1521	[M+H]+	2346827.053
Prunetin (5,4'-Dihydroxy-7-methoxyisoflavone)	552-59-0	C16H12O5	283.06	268.04	284.0685	[M-H]-	196618.125
Prolylproline	20488-28-2	C10H16N2O3	213.12	70.07	212.1161	[M+H]+	3563727.976
Procyanidin C1	37064-30-5	C45H38O18	865.2	577.14	866.2058	[M-H]-	26262.015
Procyanidin B3	23567-23-9	C30H26O12	577.14	407.08	578.1424	[M-H]-	442267.209
Procyanidin B1	20315-25-7	C30H26O12	577.14	425.09	578.1424	[M-H]-	380592.048
piscrocin C*	-	C10H12O5	211.06	148.02	212.0685	[M-H]-	291810.677
Pinoresinol-4-O-glucoside	41607-20-9	C26H32O11	519.19	357.14	520.1945	[M-H]-	981060.281
Pinoresinol-4,4'-O-diglucoside	63902-38-5	C32H42O16	681.24	519.19	682.2473	[M-H]-	1949164.736
Pinoresinol*	487-36-5	C20H22O6	357.1344	151.0384	358.1416	[M-H]-	1508854.079
Pinocembrin chalcone	4197-97-1	C15H12O4	255.07	151	256.0736	[M-H]-	125503.205
Otobaphenol*	10240-16-1	C20H22O4	327.1598	188.0828	326.1518	[M+H]+	30062543.98
otobain*	3738-01-0	C20H20O4	325.14	188.08	324.1362	[M+H]+	9190870.285
Octyl 6-O-Alpha-L-Arabinopyranosyl-Beta-D-Glucopyranoside	168288-07-1	C19H36O10	423.22	291.18	424.2308	[M-H]-	159173.853
Octadeca-9,12,15-trienoic acid	28290-79-1	C18H30O2	279.23	81.07	278.2246	[M+H]+	23422100.84
Octadec-6-enoic acid; Petroselinic acid*	593-39-5	C18H34O2	281.25	281.25	282.2559	[M-H]-	2778483.061
o-Carboxy-5-hydroxytryptamine	-	C11H12N2O3	221.09	130.06	220.0848	[M+H]+	193795.511
Nystose	13133-07-8	C24H42O21	665.21	485.15	666.2219	[M-H]-	145482.68
N-Salicyloylasparticacid	56145-94-9	C11H11NO6	252.05	136.04	253.0586	[M-H]-	20269235.42
Norisocorydine*	475-70-7	C19H21NO4	328.15	265.09	327.1471	[M+H]+	2704072.584
Norcorydine	26931-78-2	C19H21NO4	328.15	206.08	327.1471	[M+H]+	223132.07
NG,NG-Dimethyl-L-arginine*	30315-93-6	C8H18N4O2	203.15	70.07	202.143	[M+H]+	1691947.417
N-Fructosyl Pyroglutamate	-	C11H17NO8	290.09	128.04	291.0954	[M-H]-	7606942.319
N-Feruloyltyramine; Moupinamide*	66648-43-9	C18H19NO4	314.14	177.05	313.1314	[M+H]+	12185458.11
Neochlorogenic acid (5-O-Caffeoylquinic acid)*	906-33-2	C16H18O9	353.09	191.06	354.0951	[M-H]-	21122609.81
N-Cis-Feruloyltyramine*	80510-09-4	C18H19NO4	314.14	177.06	313.1314	[M+H]+	11682459.23
N-carbamoyl-b-alanine	462-88-4	C4H8N2O3	131.05	70.03	132.0535	[M-H]-	266708.024

Naringenin (5,7,4'-Trihydroxyflavanone)*	480-41-1	C15H12O5	271.06	151	272.0685	[M-H]-	98793.82
naphthisoxazol A*	-	C11H9NO2	188.07	118.06	187.0633	[M+H]+	25360441.77
N-acetyl-tryptophan	-	C13H14N2O3	245.09	203.08	246.1004	[M-H]-	67194.808
N-Acetyl-L-phenylalanine	2018-61-3	C11H13NO3	206.08	58.03	207.0895	[M-H]-	251359.911
N-Acetyl-L-glutamic acid	1188-37-0	C7H11NO5	188.06	128.04	189.0637	[M-H]-	1662369.467
N6-Threonylcarbamoyladenosine	24719-82-2	C15H20N6O8	413.14	162.04	412.1343	[M+H]+	541417.563
N6-methyladenosine	1867-73-8	C11H15N5O4	282.12	150.08	281.1124	[M+H]+	3968287.643
N5-(4-Methoxybenzyl)glutamine	-	C13H18N2O4	267.13	121.06	266.1267	[M+H]+	12193216.25
N,N'-Dimethylarginine;SDMA*	30344-00-4	C8H18N4O2	203.15	70.07	202.143	[M+H]+	1516901.195
N-(Carboxymethyl)glycine*	142-73-4	C4H7NO4	132.0303	88.0403	133.0375	[M-H]-	443203.473
N-(acetyl)phenylalanine	-	C11H13NO3	206.08	164.07	207.0895	[M-H]-	292276.525
N-(4-oxopentyl)-acetamide*	-	C7H13NO2	144.1	84.08	143.0946	[M+H]+	1450231.005
N-(1-Deoxy-1-fructosyl)Valine	-	C11H21NO7	280.14	216.12	279.1318	[M+H]+	3867622.945
N-(1-Deoxy-1-fructosyl)Phenylalanine	-	C15H21NO7	326.12	164.07	327.1313	[M-H]-	4941899.599
N-(1-Deoxy-1-fructosyl)Leucine	-	C12H23NO7	292.14	130.09	293.1475	[M-H]-	5112633.76
N-(1-Deoxy-1-fructosyl)isoleucine	-	C12H23NO7	294.15	86.1	293.1475	[M+H]+	17866956.29
Myristicanol B*	114912-33-3	C22H28O7	405.19	167.07	404.1835	[M+H]+	6738088.179
Myristargenol B	-	C20H26O5	345.17	148.05	346.178	[M-H]-	269186.646
Myrislignan	171485-39-5	C21H26O6	373.16	164.05	374.1729	[M-H]-	34744957.46
Methylmalonic acid*	516-05-2	C4H6O4	117.0193	73.0295	118.0266	[M-H]-	29770352.4
Methyleugenol*	93-15-2	C11H14O2	179.1067	123.0441	178.0994	[M+H]+	9527777.027
Methylcoclaurine*	3423-07-2	C18H21NO3	300.15	269.12	299.1521	[M+H]+	1965351.842
Methyl cumalate	6018-41-3	C7H6O4	153.02	109.03	154.0266	[M-H]-	32810768.09
Methyl 4-hydroxybenzoate	99-76-3	C8H8O3	151.04	136.02	152.0473	[M-H]-	4148574.801
Methyl 3-(3-hydroxy-4-methoxyphenyl)propanoate	129150-61-4	C11H14O4	209.08	179.03	210.0892	[M-H]-	374484.64
Methyl 2,4-dihydroxyphenylacetate*	67828-42-6	C9H10O4	181.05	135.04	182.0579	[M-H]-	3187429.944
Melibiose	585-99-9	C12H22O11	341.11	113.02	342.1162	[M-H]-	549032.433
Manninotriose	13382-86-0	C18H32O16	503.16	383.12	504.169	[M-H]-	63211.083
Maltotriose	1109-28-0	C18H32O16	527.16	365.1	504.169	[M+Na]+	422625.716
malabaricone c	63335-25-1	C21H26O5	359.18	249.15	358.178	[M+H]+	3400569.124
malabaricone b*	63335-24-0	C21H26O4	343.18	123.04	342.1831	[M+H]+	3312457.075
maingayic acid C	-	C15H22O4	265.14	122.04	266.1518	[M-H]-	76756.385
Magnocurarine	6801-40-7	C19H24NO3+	314.17	269.12	314.1751	[M]+	18600195.01
Maesopsin*	5989-16-2	C15H12O6	287.05	125.03	288.0634	[M-H]-	102511.365
Macelignan; Anwulignan	107534-93-0	C20H24O4	329.18	137.06	328.1675	[M+H]+	72234.073
LysoPC 17:2*	-	C25H48NO7P	506.32	184.07	505.3168	[M+H]+	426394.203
LysoPC 15:0*	108273-89-8	C23H48NO7P	482.32	184.07	481.3168	[M+H]+	796640.265
Lyoniside*	34425-25-7	C27H36O12	551.21	389.16	552.2207	[M-H]-	270814.001
L-Valine	72-18-4	C5H11NO2	118.09	72.08	117.079	[M+H]+	49729938.69
L-Tryptophan	73-22-3	C11H12N2O2	203.08	116.05	204.0899	[M-H]-	6758283.662
L-Seryl-L-Isoleucine	91086-51-0	C9H18N2O4	219.13	60.04	218.1267	[M+H]+	2207236.464
L-Pipecolic Acid	3105-95-1	C6H11NO2	130.09	84.08	129.079	[M+H]+	14748733.1

L-Phenylalanine	63-91-2	C9H11NO2	166.09	120.08	165.079	[M+H]+	167542907.3
L-Norleucine*	327-57-1	C6H13NO2	132.1	86.1	131.0946	[M+H]+	132418809.9
L-Malic acid*	97-67-6	C4H6O5	133.01	71.01	134.0215	[M-H]-	93238147.05
L-Leucyl-L-Leucine	3303-31-9	C12H24N2O3	245.19	86.1	244.1787	[M+H]+	5521351.565
L-Leucine*	61-90-5	C6H13NO2	132.1019	86.0964	131.0946	[M+H]+	132264580.5
L-Isoleucine*	73-32-5	C6H13NO2	132.1019	86.0964	131.0946	[M+H]+	135517317.1
Linolelaidic acid ethyl ester*	6114-21-2	C20H36O2	307.26	307.26	308.2715	[M-H]-	94649.509
Licarin B*	51020-87-2	C20H20O4	325.14	188.08	324.1362	[M+H]+	9081187.227
Licarin A*	23518-30-1	C20H22O4	327.16	188.08	326.1518	[M+H]+	31419202.34
L-Histidine	71-00-1	C6H9N3O2	156.08	110.07	155.0695	[M+H]+	2295084.447
L-Gulono-1,4-Lactone*	1128-23-0	C6H10O6	177.04	59.01	178.0477	[M-H]-	860419.278
L-Glucose*	921-60-8	C6H12O6	179.0565	59.0141	180.0634	[M-H]-	18392672.32
Leu-Pro	-	C11H20N2O3	229.15	116.07	228.1474	[M+H]+	3203934.169
L-Cyclopentylglycine	2521-84-8	C7H13NO2	144.1	98.09	143.0946	[M+H]+	1165871.74
Lauryldiethanolamine	1541-67-9	C16H35NO2	274.28	88.08	273.2668	[M+H]+	685694.423
L-Aspartyl-L-Phenylalanine	13433-09-5	C13H16N2O5	281.11	166.09	280.1059	[M+H]+	934999.12
L-Aspartic Acid*	56-84-8	C4H7NO4	132.03	88.04	133.0375	[M-H]-	4206662.98
L-Asparagine	70-47-3	C4H8N2O3	133.06	74.02	132.0535	[M+H]+	23904483.64
Lariciresinol 4-O-glucoside	143663-00-7	C26H34O11	521.2	329.14	522.2101	[M-H]-	9378706.217
L-Arginine	74-79-3	C6H14N4O2	175.12	116.07	174.1117	[M+H]+	31665338.77
L-Alanyl-L-Phenylalanine	3061-90-3	C12H16N2O3	237.12	120.09	236.1161	[M+H]+	1337616.81
Koaburaside	41653-73-0	C14H20O9	331.1	153.02	332.1107	[M-H]-	874664.039
Kadsuranin*	82467-51-4	C23H28O6	401.19	193.09	400.1886	[M+H]+	57977812.75
Isomaltulose*	13718-94-0	C12H22O11	341.1089	89.0249	342.1162	[M-H]-	2783881.209
Isolariciresinol-9'-O-glucoside*	63358-12-3	C26H34O11	521.2	359.15	522.2101	[M-H]-	1793933.934
Isohomogenol*	93-16-3	C11H14O2	179.11	123.04	178.0994	[M+H]+	8955442.392
Isogalcatin	-	C21H24O4	341.17	178.1	340.1675	[M+H]+	8115438.429
Isoferulic Acid*	25522-33-2	C10H10O4	193.0506	134.0374	194.0579	[M-H]-	9359206.634
Isocitric Acid	320-77-4	C6H8O7	191.02	111.01	192.027	[M-H]-	12241919.54
Isochlorogenic acid C*	57378-72-0	C25H24O12	515.12	353.09	516.1268	[M-H]-	12260963.27
Isochlorogenic acid B	14534-61-3	C25H24O12	515.12	353.09	516.1268	[M-H]-	12490269.73
Isochlorogenic acid A*	2450-53-5	C25H24O12	515.12	353.09	516.1268	[M-H]-	11710659.9
Isoboldine	3019-51-0	C19H21NO4	328.16	265.09	327.1471	[M+H]+	3630477.989
Icariside E5*	126176-79-2	C26H34O11	521.2	359.15	522.2101	[M-H]-	757310.485
Hydroxytyrosol	10597-60-1	C8H10O3	153.06	123.05	154.063	[M-H]-	1420674.636
hydroxyotobain	-	C20H20O5	339.12	324.1	340.1311	[M-H]-	1659424.934
Hydroxy ricinoleic acid	-	C18H34O4	313.24	183.14	314.2457	[M-H]-	4973510.33
Hydroperoxylinoleic acid*	34444-18-3	C18H32O4	311.22	223.17	312.2301	[M-H]-	1009936.773
Hydrohydrastinine*	494-55-3	C11H13NO2	192.1	177.08	191.0946	[M+H]+	32606975.4
Homoproline	56879-46-0	C6H11NO2	130.09	70.07	129.079	[M+H]+	44241591.97
Higenamine*	5843-65-2	C16H17NO3	272.12	107.05	271.1208	[M+H]+	4085252.636
Hexadecanedioic acid	505-54-4	C16H30O4	285.21	223.21	286.2144	[M-H]-	1524480.715
Hercynine	534-30-5	C9H15N3O2	198.12	95.06	197.1164	[M+H]+	4039165.497

Heminordihydroguaiaretic acid	54473-24-4	C19H24O4	315.16	149.06	316.1675	[M-H]-	526649.027
Gypensapogenin F*	-	C30H48O4	471.34	471.34	472.3553	[M-H]-	404634.215
Gusanlung B	79082-05-6	C20H19NO5	354.13	339.11	353.1263	[M+H]+	5061739.634
Guanosine 3',5'-cyclic monophosphate	7665-99-8	C10H12N5O7P	344.04	150.04	345.0474	[M-H]-	832995.318
Guanosine	118-00-3	C10H13N5O5	284.1	152.06	283.0917	[M+H]+	82402515.95
Guanine	73-40-5	C5H5N5O	152.06	135.03	151.0494	[M+H]+	4111376.723
Guaiacylglycerol-β-Guaiacyl Ether	7382-59-4	C17H20O6	319.12	241.05	320.126	[M-H]-	185850.322
Gomisin N*	69176-52-9	C23H28O6	401.2	193.09	400.1886	[M+H]+	59102282.06
Gnetifolin B*	140671-06-3	C16H12O6	299.06	284.03	300.0634	[M-H]-	136806.758
Glucosyloxybenzoic acid*	-	C13H16O8	299.08	137.02	300.0845	[M-H]-	1870593.794
Gluconic acid	526-95-4	C6H12O7	195.05	75.01	196.0583	[M-H]-	3708413.117
Glucan	9041-22-9	C18H32O16	505.17	145.05	504.169	[M+H]+	115869.81
Gallic Acid 4-O-Glucoside	-	C13H16O10	331.07	168.01	332.0743	[M-H]-	4097990.334
Gallic acid	149-91-7	C7H6O5	169.01	125.02	170.0215	[M-H]-	3452263.821
Galcatin	-	C21H24O4	341.18	188.08	340.1675	[M+H]+	4171605.881
fragransin D1	114394-21-7	C22H28O6	389.2	181.08	388.1886	[M+H]+	9193446.891
fragransin C1	-	C21H26O6	373.17	194.06	374.1729	[M-H]-	3955697.475
Fragransin B3	-	C22H28O7	405.19	251.12	404.1835	[M+H]+	1751581.03
Fragransin B1*	-	C22H28O7	405.19	167.07	404.1835	[M+H]+	7026682.314
fragransin A2*	-	C20H24O5	345.1717	137.0633	344.1624	[M+H]+	23892828.23
Flavokawain B*	1775-97-9	C17H16O4	285.11	181.05	284.1049	[M+H]+	1151994.541
Feruloyloctose*	-	C18H24O11	415.12	193.05	416.1319	[M-H]-	1831274.573
Ferulic acid-4-O-glucoside	117405-51-3	C16H20O9	355.1	193.05	356.1107	[M-H]-	378349.829
Ferulic acid*	537-98-4	C10H10O4	193.05	134.04	194.0579	[M-H]-	7275175.341
Ferulaldehyde	-	C10H10O3	177.06	162.03	178.063	[M-H]-	6159903.992
Ethyl ferulate	4046-02-0	C12H14O4	221.09	133.03	222.0892	[M-H]-	768793.289
Ethyl caffeate	102-37-4	C11H12O4	207.07	135.04	208.0736	[M-H]-	1249127.891
Ethyl (10Z,13Z)-hexadeca-10,13-dienoate	-	C18H32O2	279.23	261.23	280.2402	[M-H]-	95652.482
Esculetin (6,7-Dihydroxycoumarin)	305-01-1	C9H6O4	177.02	133.03	178.0266	[M-H]-	967389.401
Epipinoresinol*	24404-50-0	C20H22O6	357.13	151.04	358.1416	[M-H]-	1348627.994
Emodin	518-82-1	C15H10O5	269.05	225.06	270.0528	[M-H]-	190580.479
Elemicin	487-11-6	C12H16O3	209.11	153.05	208.1099	[M+H]+	99717167.94
Elaidic Acid*	112-79-8	C18H34O2	281.25	281.25	282.2559	[M-H]-	7685049.417
Eicosadienoic acid*	5598-38-9	C20H36O2	307.2643	307.2643	308.2715	[M-H]-	254195.182
Ebracteatoside D	-	C19H34O11	497.22	305.16	438.2096	[M+CH3COO]-	82446.989
D-Xylonic acid*	526-91-0	C5H10O6	165.04	75.01	166.0477	[M-H]-	5075329.862
D-Trehalose*	99-20-7	C12H22O11	341.11	119.03	342.1162	[M-H]-	527482.302
D-Threonic Acid	3909-12-4	C4H8O5	135.03	75.01	136.0372	[M-H]-	1272456.065
D-Sucrose*	57-50-1	C12H22O11	341.11	119.03	342.1162	[M-H]-	1253557.234
D-Saccharic acid	87-73-0	C6H10O8	209.03	85.03	210.0376	[M-H]-	190939.171
Drimiopsin C*	773850-90-1	C15H12O6	287.05	125.02	288.0634	[M-H]-	56035.11
D-Pantothenic Acid 4'-O-Beta-Glucoside	29588-37-2	C15H27NO10	382.17	220.12	381.1635	[M+H]+	3430720.495
D-Pantothenic Acid	79-83-4	C9H17NO5	220.12	90.05	219.1107	[M+H]+	8704663.058

D-Panose*	33401-87-5	C18H32O16	503.16	179.06	504.169	[M-H]-	1005487.955
Dodecanoic acid (Lauric acid)	143-07-7	C12H24O2	199.17	199.17	200.1776	[M-H]-	1129616.621
Dodecanedioic aicd	693-23-2	C12H22O4	229.14	211.13	230.1518	[M-H]-	14515.987
D-Mannose*	3458-28-4	C6H12O6	179.06	59.01	180.0634	[M-H]-	21849116.78
D-Maltotetraose	34612-38-9	C24H42O21	665.21	161.04	666.2219	[M-H]-	84900.776
D-Maltose*	69-79-4	C12H22O11	341.1089	89.0244	342.1162	[M-H]-	2697294.843
D-Malic acid*	636-61-3	C4H6O5	133.01	71.01	134.0215	[M-H]-	24114021.54
DL-Tryptophan	54-12-6	C11H12N2O2	205.09	146.06	204.0899	[M+H]+	106498428.2
DL-Leucine*	328-39-2	C6H13NO2	132.1	86.1	131.0946	[M+H]+	29720217.72
D-Lactose*	63-42-3	C12H22O11	341.11	89.02	342.1162	[M-H]-	3366128.068
DL-2-hydroxystearic acid*	629-22-1	C18H36O3	299.26	253.25	300.2664	[M-H]-	49851.22
Dihydroberberine*	-	C20H19NO4	338.14	323.12	337.1314	[M+H]+	53272512.96
Dihomocitric acid	-	C8H12O7	219.05	111.01	220.0583	[M-H]-	3468059.019
DGMG 18:3	-	C33H56O14	735.38	397.13	676.367	[M+CH3COO]-	199461.768
D-Glucose-1-phosphate*	59-56-3	C6H13O9P	259.02	96.97	260.0297	[M-H]-	888868.041
D-Glucose*	50-99-7	C6H12O6	179.0561	59.0139	180.0634	[M-H]-	18881826.91
D-Glucose 6-phosphate*	56-73-5	C6H13O9P	259.0225	96.9696	260.0297	[M-H]-	902556.653
D-Glucono-1,5-lactone*	90-80-2	C6H10O6	177.0449	59.0139	178.0477	[M-H]-	1122268.987
D-Galactose*	59-23-4	C6H12O6	179.06	59.01	180.0634	[M-H]-	15492494.73
D-Galactaric acid	526-99-8	C6H10O8	209.03	85.03	210.0376	[M-H]-	341312.837
D-Fructose*	57-48-7	C6H12O6	179.06	59.01	180.0634	[M-H]-	18113693.38
D-Fructose 6-Phosphate*	643-13-0	C6H13O9P	259.02	96.97	260.0297	[M-H]-	734084.174
Demethylcoclaurine*	106032-53-5	C16H17NO3	272.13	107.05	271.1208	[M+H]+	3003629.802
Dehydrophytosphingosine	-	C18H37NO3	316.29	60.04	315.2773	[M+H]+	2372677.245
Dehydrodiisoeugenol*	2680-81-1	C20H22O4	327.1523	188.0792	326.1518	[M+H]+	31220787.94
Dehydrocorydaline	30045-16-0	C22H24NO4+	366.17	351.15	366.17	[M]+	4248979.017
D-Arabinono-1,4-lactone	2782-09-4	C5H8O5	147.03	85.03	148.0372	[M-H]-	175197.657
D-Arabinoic acid*	32609-14-6	C5H10O6	165.04	75.01	166.0477	[M-H]-	2017435.225
D-Allo-Isoleucine*	1509-35-9	C6H13NO2	132.1	86.1	131.0946	[M+H]+	131238387.1
Cytidine	65-46-3	C9H13N3O5	244.09	112.05	243.0855	[M+H]+	16906929.87
Cytarabine	147-94-4	C9H13N3O5	244.09	112.05	243.0855	[M+H]+	3148410.074
Cycloolivil-6-O-glucoside	-	C26H34O12	537.2	375.15	538.205	[M-H]-	649870.562
Cyclo(Pro-Pro)	6708-06-1	C10H14N2O2	195.11	70.06	194.1055	[M+H]+	1060112.285
C-Veratroylglycol*	168293-10-5	C10H12O5	211.0601	148.017	212.0685	[M-H]-	236743.018
Cryptochlorogenic acid (4-O-Caffeoylquinic acid)*	905-99-7	C16H18O9	353.09	191.06	354.0951	[M-H]-	34998090.46
Cordycepin (3'-Deoxyadenosine)*	73-03-0	C10H13N5O3	252.11	136.06	251.1018	[M+H]+	4359303.244
Cordianal C*	-	C30H48O4	471.35	471.35	472.3553	[M-H]-	252154.164
Coniferyl alcohol	458-35-5	C10H12O3	179.07	146.04	180.0786	[M-H]-	481714.418
Codeinone	467-13-0	C18H19NO3	298.14	283.12	297.1365	[M+H]+	4503849.219
Coclaurine*	15548-30-8	C17H19NO3	286.14	107.05	285.1365	[M+H]+	4479407.504
Citric Acid	77-92-9	C6H8O7	191.02	111.01	192.027	[M-H]-	128331553.5
Cimidahurinine*	142542-89-0	C14H20O8	315.11	153.06	316.1158	[M-H]-	54261419.26

Choline Alfoscerate	28319-77-9	C8H20NO6P	258.11	104.11	257.1028	[M+H]+	2104643.344
Chlorogenic acid (3-O-Caffeoylquinic acid)*	327-97-9	C16H18O9	353.09	191.06	354.0951	[M-H]-	18554277.09
Catechin 4'-O-beta-D-glucopyranoside*	-	C21H24O11	451.12	289.07	452.1319	[M-H]-	542771.889
Caffeic acid 4-O-glucoside	14364-08-0	C15H18O9	341.09	161.02	342.0951	[M-H]-	1633966.93
Caffeic acid	331-39-5	C9H8O4	179.03	135.05	180.0423	[M-H]-	13996099.58
Beta-Hydroxypalmitic Acid*	-	C16H32O3	271.23	225.22	272.2351	[M-H]-	6920597.177
Benzhydryl methyl ether*	1016-09-7	C14H14O	197.1	182.07	198.1045	[M-H]-	542834.2
Azelaic acid	123-99-9	C9H16O4	187.1	125.1	188.1049	[M-H]-	12851645.11
Argemonine	6901-16-2	C21H25O4N	356.19	311.13	355.1784	[M+H]+	17760715.41
Arbutin	497-76-7	C12H16O7	271.08	108.02	272.0896	[M-H]-	24529.02
Arachidonic Acid	506-32-1	C20H32O2	303.23	259.24	304.2402	[M-H]-	30165.204
alpha-Hydroxylinoleic acid*	57818-44-7	C18H32O3	295.23	195.14	296.2351	[M-H]-	12138427.22
alanine betaine	-	C5H11NO2	118.08	72.08	117.079	[M+H]+	13640198.11
Adenosine*	58-61-7	C10H13N5O4	268.1	136.06	267.0968	[M+H]+	136380369.9
9S-Hydroxy-10E,12Z-octadecadienoic acid*	15514-85-9	C18H32O3	295.23	195.14	296.2351	[M-H]-	13043576.05
9-Oxo-10,12-Octadecadienoic Acid	54665-32-6	C18H30O3	295.23	151.11	294.2195	[M+H]+	6228474.653
9-Hydroxy-12-oxo-15(Z)-octadecenoic acid*	-	C18H32O4	311.2228	223.1717	312.2301	[M-H]-	1016536.501
9-Arabinosyladenine*	-	C10H13N5O4	268.11	136.07	267.0968	[M+H]+	131760874.1
9-Alpha-Ribofuranosyladenine*	-	C10H13N5O4	268.1	136.06	267.0968	[M+H]+	159736470.2
9,12,13-Trihydroxy-10,15-octadecadienoic acid	-	C18H32O5	327.22	291.2	328.225	[M-H]-	781351.232
9,12,13-TriHOME; 9(S),12(S),13(S)-Trihydroxy-10(E)-octadecenoic acid	97134-11-7	C18H34O5	329.23	211.14	330.2406	[M-H]-	9684840.428
9,10,13-Trihydroxy-11-Octadecenoic Acid	29907-57-1	C18H34O5	329.23	229.15	330.2406	[M-H]-	20370156.17
9,10,11-Trihydroxy-12-octadecenoic acid	61911-67-9	C18H34O5	329.23	311.22	330.2406	[M-H]-	1675472.329
8-Oxyberberine	549-21-3	C20H17NO5	352.12	337.1	351.1107	[M+H]+	85424305.51
8-Hydroxypinoresinol 4-Glucoside*	-	C26H32O12	535.18	373.13	536.1894	[M-H]-	3036683.779
8-Hydroxydihydrochelerythrine	4070-42-2	C21H19NO5	366.13	351.11	365.1263	[M+H]+	235268.406
8'-epi-aristoligone	-	C22H26O5	371.19	195.1	370.178	[M+H]+	1516224.5
8,11-Heptadecadienoic acid	-	C17H30O2	265.22	265.22	266.2246	[M-H]-	150348.579
7-methoxy-1-[(4-methoxyphenyl)methyl]-2-methyl-3,4-dihydro-1h-isoquinolin-6-ol	-	C19H23NO3	314.18	107.05	313.1678	[M+H]+	15803310.61
7-Hydroxycoumarin;Umbelliferone	93-35-6	C9H6O3	161.02	133.03	162.0317	[M-H]-	2439294.953
7-Caffeoylsedoheptulose	-	C16H20O10	371.1	135.05	372.1056	[M-H]-	44532.5
6-Aminocaproic acid	60-32-2	C6H13NO2	132.1	69.07	131.0946	[M+H]+	23996243.05
6-[(2r,3r,4r,5s)-5-(3,4-dimethoxyphenyl)-3,4-dimethyloxolan-2-yl]-2,3-dihydro-1,4-benzodioxine	-	C22H26O5	371.19	193.09	370.178	[M+H]+	77522128.07
6-(hydroxymethyl)pyridin-3-ol	40222-77-3	C6H7NO2	126.06	80.05	125.0477	[M+H]+	3009452.738
5-O-Galloyl-D-hamamelose*	-	C13H16O10	331.07	169.02	332.0743	[M-H]-	79532.004
5-O-Caffeoylshikimic acid	73263-62-4	C16H16O8	335.08	135.05	336.0845	[M-H]-	1834352.187
5-O-(3'-O-Glucosylcaffeoyl)Quinic Acid	1629852-63-6	C22H28O14	515.14	179.04	516.1479	[M-H]-	78972.382
5-Methoxytryptophol*	712-09-4	C11H13NO2	192.1	177.08	191.0946	[M+H]+	1030170.581

5'-Deoxyadenosine*	4754-39-6	C10H13N5O3	252.1	136.06	251.1018	[M+H]+	5582650.127
5-Aminoimidazole ribonucleotide	25635-88-5	C8H14N3O7P	296.07	104.11	295.0569	[M+H]+	1526239.185
5-Allyl-1,3-dimethoxy-2-((1-(3,4,5-trimethoxyphenyl)propan-2-yl)oxy)benzene*	-	C23H30O6	403.21	209.12	402.2042	[M+H]+	5637907.193
5-{[2-O-(beta-d-apiofuranosyl)-beta-d-glucopyranosyl]oxy}-2-hydroxybenzoic acid*	-	C18H24O13	447.11	152.01	448.1217	[M-H]-	73893.997
5,8-dimethoxy-3-methylnaphthalen-1-ol	50559-08-5	C13H14O3	219.1	189.09	218.0943	[M+H]+	756535.717
4-O-Feruloylquinic acid	2613-86-7	C17H20O9	367.1	193.05	368.1107	[M-H]-	92045202.58
4-Methylazetidine-2-Carboxylic acid	1779725-63-1	C5H9NO2	116.07	70.06	115.0633	[M+H]+	178997104.1
4-Methoxyphenylpropionic acid	-	C10H12O3	179.07	146.04	180.0786	[M-H]-	82114.924
4-Methoxydiphenylmethane*	834-14-0	C14H14O	197.1	182.07	198.1045	[M-H]-	525340.098
4-ketopinoresinol	66288-89-9	C20H20O7	371.11	282.09	372.1209	[M-H]-	267253.583
4-Hydroxyphenyllactic Acid*	306-23-0	C9H10O4	181.05	135.04	182.0579	[M-H]-	3953755.068
4-Hydroxybenzaldehyde	123-08-0	C7H6O2	121.03	92.03	122.0368	[M-H]-	6074380.418
4-Guanidinobutyric acid	463-00-3	C5H11N3O2	146.09	87.04	145.0851	[M+H]+	9071444.848
4-caffeoylshikimic acid*	-	C16H16O8	335.08	179.03	336.0845	[M-H]-	3806323.09
4-[4-(4-hydroxy-3-methoxyphenyl)-2,3-dimethylbutyl]-2-methoxyphenol	36469-60-0	C20H26O4	329.17	314.15	330.1831	[M-H]-	1718330.327
4,5-Dehydro-L-leucine	-	C6H11NO2	130.09	84.08	129.079	[M+H]+	16398267.74
3-Oxoolean-12-en-28-oic Acid (Oleanonic acid)*	17990-42-0	C30H46O3	453.34	453.34	454.3447	[M-H]-	30191.323
3-Oxo-9,19-cyclolanost-24-en-26-oic acid (Mangiferonic acid)*	13878-90-5	C30H46O3	453.34	453.34	454.3447	[M-H]-	11337.896
3-O-Galloyl-D-glucose*	-	C13H16O10	331.07	169.01	332.0743	[M-H]-	100073.683
3-O-Feruloylquinic acid	1899-29-2	C17H20O9	367.1	193.05	368.1107	[M-H]-	105789886.1
3-O-Feruloyl Octose*	-	C18H24O11	415.12	193.05	416.1319	[M-H]-	1972259.235
3-O-caffeoylshikimic acid*	-	C16H16O8	335.0756	179.0343	336.0845	[M-H]-	3273061
3-Isopropylmalic Acid*	921-28-8	C7H12O5	175.0612	115.0399	176.0685	[M-H]-	6787810.116
3-Indoleacrylic acid*	1204-06-4	C11H9NO2	188.0706	118.0651	187.0633	[M+H]+	51938039.36
3-Hydroxy-3-methylpentane-1,5-dioic acid	503-49-1	C6H10O5	161.05	99.05	162.0528	[M-H]-	2869969.702
3-Dehydro-L-Threonic Acid	-	C4H6O5	133.01	71.01	134.0215	[M-H]-	30944362.76
3-amino-2-naphthoic acid*	-	C11H9NO2	188.07	118.06	187.0633	[M+H]+	23134902.18
3,7-Dimethylocta-2,6-Diene-1,4-Diol 1-O-Beta-D-Glucopyranoside	-	C16H28O7	391.2	161.05	332.1835	[M+CH3COO]-	226188.515
3,5-Dicaffeoylquinic acid	-	C25H24O12	515.12	353.09	516.1268	[M-H]-	1316949.943
3,5,7-Trimethoxyflavone	26964-29-4	C18H16O5	313.11	297.08	312.0992	[M+H]+	1431445.83
3,5,7-Trihydroxyflavanone (Pinobanksin)*	548-82-3	C15H12O5	271.0612	151.0032	272.0685	[M-H]-	63712.292
3,4-Dihydroxybenzoic Acid Ethyl Ester (Protocatechuic acid ethyl ester)	3943-89-3	C9H10O4	181.05	109.03	182.0579	[M-H]-	3135609.42
3,4-Dihydroxybenzoic acid (Protocatechuic acid)*	99-50-3	C7H6O4	153.02	109.03	154.0266	[M-H]-	66873223.16
3',4'-Dihydroxyacetophenone	1197-09-7	C8H8O3	151.04	108.02	152.0473	[M-H]-	290186.273

3,3-bis-(4-hydroxy-3-methoxyphenyl)-propane-1,2-diol	-	C17H20O6	319.12	241.05	320.126	[M-H]-	209613.188
3,23-Dihydroxyolean-12-en-28-oic acid (Hederagenin)*	465-99-6	C30H48O4	471.3514	471.3514	472.3553	[M-H]-	741676.205
3,19-Dihydroxyurs-12-en-28-oic acid (Pomolic acid)*	13849-91-7	C30H48O4	471.35	471.35	472.3553	[M-H]-	1164553.197
3-(Hydroxycinnamoyl)-quinic acid*	-	C16H18O8	337.09	163.04	338.1002	[M-H]-	1320545.544
2-Propylmalic Acid*	-	C7H12O5	175.06	115.04	176.0685	[M-H]-	5919838.664
2-Phenylethyl beta-primeveroside	129932-48-5	C19H28O10	415.16	89.02	416.1682	[M-H]-	201670.152
2'-O-Methyladenosine	2140-79-6	C11H15N5O4	282.12	136.06	281.1124	[M+H]+	9664383.921
2-Methyl-3,4-dihydropapaverinium	96550-47-9	C21H26NO4+	356.19	206.12	356.1862	[M]+	4385637.767
2-Linoleoylglycerol*	3443-82-1	C21H38O4	355.28	263.24	354.277	[M+H]+	215207.487
2-Isopropylmalic Acid	49601-06-1	C7H12O5	175.06	115.04	176.0685	[M-H]-	6407355.953
2-Hydroxyursolic acid*	-	C30H48O4	471.35	471.35	472.3553	[M-H]-	281157.413
2-Hydroxymyristic acid	2507-55-3	C14H28O3	243.2	197.19	244.2038	[M-H]-	363093.022
2-Hydroxyhexadecanoic acid*	764-67-0	C16H32O3	271.23	225.22	272.2351	[M-H]-	7334150.136
2-Hydroxy-3-(4-Hydroxyphenyl)Propanoic Acid*	23508-35-2	C9H10O4	181.05	135.04	182.0579	[M-H]-	3625410.171
2'-Deoxyadenosine*	958-09-8	C10H13N5O3	252.11	136.06	251.1018	[M+H]+	7728841.669
2-Aminopurine	452-06-2	C5H5N5	136.06	119.04	135.0545	[M+H]+	41694330.59
23-Hydroxy-3-oxoolean-12-en-28-oic acid (Hederagonic acid)	466-01-3	C30H46O4	469.33	439.32	470.3396	[M-H]-	8429.842
2-[4-(3-Hydroxypropyl)-2-methoxyphenoxy]-1,3-propanediol 1-glucoside	-	C19H30O10	477.2	181.09	418.1839	[M+CH3COO]-	368239.417
2,5-Dihydroxybenzoic acid; Gentisic Acid*	490-79-9	C7H6O4	153.02	109.03	154.0266	[M-H]-	30542863.87
2,3-Dihydroxyurs-12-en-28-oic acid (Corosolic acid)*	4547-24-4	C30H48O4	471.3482	471.348	472.3553	[M-H]-	637843.973
2,3-Dihydroxyolean-12-en-28-oic acid (2-Hydroxyoleanolic acid)*	26707-60-8	C30H48O4	471.35	471.35	472.3553	[M-H]-	226300.326
2,3-Dihydroxylup-20(29)-en-28-oic acid (Alphitolic acid)*	19533-92-7	C30H48O4	471.348	471.348	472.3553	[M-H]-	255987.732
2,3-Dihydroxybenzoic Acid*	303-38-8	C7H6O4	153.0193	109.0295	154.0266	[M-H]-	69724357.7
2,3-Dihydroxy-12-ursen-28-oic acid*	-	C30H48O4	471.348	471.34	472.3553	[M-H]-	858791.926
2,3-Dihydroxy-1-(4'-hydroxy-3'-methoxyphenyl)-propan-1-one*	-	C10H12O5	211.06	148.02	212.0685	[M-H]-	312517.783
2,2'-(3-methylcyclohexane-1,1-diyl)diacetic acid	5345-12-0	C11H18O4	213.11	169.12	214.1205	[M-H]-	113969.08
1-O-(3,4,5-Trimethoxybenzoyl)-B-D-Glucopyranoside	-	C16H22O10	373.11	211.06	374.1213	[M-H]-	363257.45
1-Monomyristin	589-68-4	C17H34O4	303.25	57.07	302.2457	[M+H]+	575775.858
1-Methylpiperidine-2-carboxylic acid*	7730-87-2	C7H13NO2	144.1019	84.0813	143.0946	[M+H]+	1505952.894
1-Linoleoylglycerol*	2277-28-3	C21H38O4	355.28	263.24	354.277	[M+H]+	215230.155
1-Hydroxypinoresinol-1-O-Glucoside*	81495-71-8	C26H32O12	535.18	373.13	536.1894	[M-H]-	2681043.003
1-Caffeoylquinic acid	1241-87-8	C16H18O9	353.09	191.06	354.0951	[M-H]-	25196075.05

16,23:16,30-Diepoxydammar-24-ene-3,20-diol (Jujubogenin)*	54815-36-0	C30H48O4	471.35	471.34	472.3553	[M-H]-	551232.438
13-methylmyristic acid	2485-71-4	C15H30O2	241.22	241.21	242.2246	[M-H]-	68321.606
13-Amino-13-oxotridecanoic acid	-	C13H25NO3	242.18	225.15	243.1834	[M-H]-	69345.981
13(S)-HODE;13(S)-Hydroxyoctadeca-9Z,11E-dienoic acid*	10219-69-9	C18H32O3	295.23	195.14	296.2351	[M-H]-	7273519.564
12-Hydroxyjasmonic Acid Glucoside	124649-25-8	C18H28O9	387.16	59.01	388.1733	[M-H]-	46704
10-Formyltetrahydrofolic Acid	2800-34-2	C20H23N7O7	474.17	327.12	473.1659	[M+H]+	6580506.833
1-[(4-methoxyphenyl)methyl]-1,2,3,4-tetrahydroisoquinoline-6,7-diol*	-	C17H19NO3	286.14	107.05	285.1365	[M+H]+	4099458.509
1,4-bis(3,4-dimethoxyphenyl)-2,3-dimethylbutan-1-one	-	C22H28O5	373.2	181.08	372.1937	[M+H]+	2820317.866
1,2,10-Trimethoxy-3,9-Dihydroxy-Aporphine	-	C20H23NO5	358.16	58.06	357.1576	[M+H]+	1613318.037
1-(4-hydroxyphenyl)-7-methoxy-1,2,3,4-tetrahydroisoquinolin-8-ol*	-	C16H17NO3	272.13	107.05	271.1208	[M+H]+	4174381.979
1-(2,6-dihydroxyphenyl)-9-［4-hydroxy-3-(p-menth-1-en-8-oxy)-phenyl］-1-nonanone	-	C31H42O5	493.3	357.17	494.3032	[M-H]-	1569963.583
1-(2,6-dihydroxyphenyl)-9-(4-hydroxyphenyl)-1-nonanone*	-	C21H26O4	343.19	123.05	342.1831	[M+H]+	3290779.449
[(2S,3S)-2-(3,4-dimethoxyphenyl)-7-methoxy-5-prop-2-enyl-2,3-dihydro-1-benzofuran-3-yl]methanol	-	C21H24O5	357.17	151.07	356.1624	[M+H]+	5690393.217
(S)-Malic acid-1-O-beta-D-glucopyranoside	-	C10H16O10	295.07	115	296.0743	[M-H]-	269301.132
(E)-caffeyl alcohol 4-O-β-D-glucopyranoside	-	C15H20O8	327.11	147.05	328.1158	[M-H]-	166355.12
(9Z,11E)-Octadecadienoic acid	2540-56-9	C18H32O2	279.233	59.0133	280.2402	[M-H]-	8434.989
(9S)-Hydroxyoctadecadienoic acid	73543-67-6	C18H32O3	295.23	171.1	296.2351	[M-H]-	12670476.24
(1-Methylpyrrolidin-2-yl)aceticacid*	5626-43-7	C7H13NO2	144.1	84.08	143.0946	[M+H]+	1463001.874
