# Supplementary material for: Xu Chunfu’s Modified Xianglian Pill Regulates the NOX2/ROS/Mitochondria/NLRP3 Axis to Treat Ulcerative Colitis
Source: Pharmaceuticals (Basel). 2026 Mar 11;19(3):452. doi: 10.3390/ph19030452 (PMC13029697; doi:10.3390/ph19030452)
Supplement: Supplementary file 1 [file pharmaceuticals-19-00452-s001.zip › pharmaceuticals-4158266-Table S2.pdf]

Supplementary Table S2 Antibody dilutions.

Antibody Name	Antibody Brand	Catalog	Antibody dilution ratio
NOX2	Abclonal	A19701	1:1000
CYBA	Abclonal	A10694	1:1000
p67	Abclonal	A1178	1:1000
Phospho-p47	Affinity	AF3917	1:200
p47	Abclonal	A1148	1:500
p40	Abclonal	A2096	1:1000
S100A8	Abclonal	A1688	1:500
S100A9	Abclonal	A9842	1:500
ASC	Abclonal	A22046	1:1000
Cleaved Caspase-1	Abclonal	A23429	1:500
Rac2	Proteintech	60077-1-Ig	1:1000
NLRP3	Cell Signaling Technology	15101T	1:1000
Caspase-1	Cell Signaling Technology	83383T	1:1000
β-actin	HUABIO	R1207-1	1:5000
