# Supplementary material for: Xu Chunfu’s Modified Xianglian Pill Regulates the NOX2/ROS/Mitochondria/NLRP3 Axis to Treat Ulcerative Colitis
Source: Pharmaceuticals (Basel). 2026 Mar 11;19(3):452. doi: 10.3390/ph19030452 (PMC13029697; doi:10.3390/ph19030452)
Supplement: Supplementary file 1 [file pharmaceuticals-19-00452-s001.zip › pharmaceuticals-4158266-Table S3.pdf]

Supplementary Table S3 Primers used in the quantity real-time PCR assay.

Genes	Species	Primer sequence (5' to 3')	
Cybb	mouse	Forward	AGCAACAGAAGAAGAAGCACAGTAG
		Reverse	CAACCACACCAGAATGACAAAGATG
Cyba	mouse	Forward	GCACCATCAAGCAACCACCTAC
		Reverse	GCCTCCTCTTCACCCTCACTC
Ncf4	mouse	Forward	ATCTCATCTACCGCCGCTATCG
		Reverse	GAAAGGGCTGTTCTTGCTCTCTG
Rac2	mouse	Forward	GACCTCAGACCTGCCATCCTC
		Reverse	ACCACATCAACTTCCATAGAACTCTC
Nlrp3	mouse	Forward	TCGCCCAAGGAGGAAGAAGAAG
		Reverse	TGAGGCAGCAGTTCACCAGTC
Asc	mouse	Forward	AGACTTTGGGCTCTGGGAACTC
		Reverse	TGGGCTGGGCATGGTGATAC
Caspase-1	mouse	Forward	AATCTGTATTCACGCCCTGTTGG
		Reverse	AATTGCTTCCTCTTTGCCCTCAG
β-actin	mouse	Forward	ACTGCCGCATCCTCTTCCTC
		Reverse	AACCGCTCGTTGCCAATAGTG
CYBB	human	Forward	AAGTGCCCAAAGGTGTCCAA
		Reverse	CCCAACGATGCGGATATGGA
CYBA	human	Forward	AGAAGTACATGACCGCCGTG
		Reverse	CAGCCGCCAGTAGGTAGATG
NCF4	human	Forward	TCTCAGCAGCACTCCCCTAT
		Reverse	CTACGTCCTCATCCGACAGC
RAC2	human	Forward	CTCCCCTCTGGAAACTGCAG
		Reverse	TCAGGAGGGAGTAAGAGCCC
iNOS	human	Forward	GGACCACATCTACCAGGAGGAG
		Reverse	CCAGGCAGGCGGGAATAGG
GAPDH	human	Forward	ACACCCACTCCTCCACCTTTG
		Reverse	TCCACCACCCTGTTGCTGTAG
