# Supplementary figures and images for: Xu Chunfu’s Modified Xianglian Pill Regulates the NOX2/ROS/Mitochondria/NLRP3 Axis to Treat Ulcerative Colitis
Source: Pharmaceuticals (Basel). 2026 Mar 11;19(3):452. doi: 10.3390/ph19030452 (PMC13029697; doi:10.3390/ph19030452)

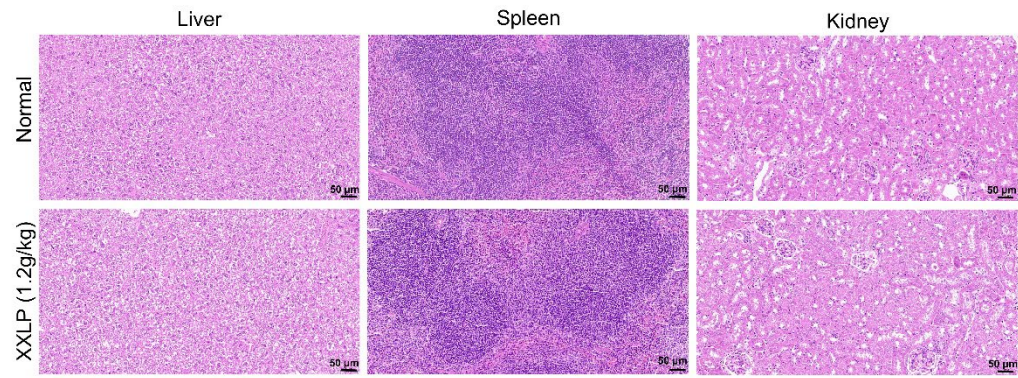

**Supplementary Figure S1: H&E staining of liver, spleen, and kidney tissues.**

Supplement: Supplementary file 1 [file pharmaceuticals-19-00452-s001.zip › pharmaceuticals-4158266-Supplementary Figure S1.pdf]
